# Pituitary Action of E2 in Prepubertal Grass Carp: Receptor Specificity and Signal Transduction for Luteinizing Hormone and Follicle-Stimulating Hormone Regulation

**DOI:** 10.3389/fendo.2018.00308

**Published:** 2018-06-08

**Authors:** Xiangfeng Qin, Yaqian Xiao, Cheng Ye, Jingyi Jia, Xiangjiang Liu, Hongwei Liang, Guiwei Zou, Guangfu Hu

**Affiliations:** ^1^College of Fisheries, Huazhong Agricultural University, Wuhan, China; ^2^Key Laboratory of Freshwater Biodiversity Conservation Ministry of Agriculture, Yangtze River Fisheries Research Institute, The Chinese Academy of Fishery Sciences, Wuhan, China

**Keywords:** E2, luteinizing hormone, follicle-stimulating hormone, growth regulation by estrogen in breast cancer 1, pituitary, grass carp

## Abstract

17β-estradiol (E2) is an important sex steroid produced by ovary and brain. In mammals, E2 plays an important role in hypothalamus–pituitary–gonad axis to regulate puberty onset, however, little is known about the functional role of E2 in teleost pituitary. Using prepubertal grass carp as model, three nuclear estrogen receptors (nERs: estrogen receptor alpha, estrogen receptor beta 1, and estrogen receptor beta 2) and two G protein-coupled estrogen receptors (GPER1: GPER1a and GPER1b) were isolated from grass carp pituitary. Tissue distribution analysis indicated that both nERs and GPERs were highly detected in grass carp pituitary, which suggested that E2 should play an important role in grass carp pituitary. Using primary cultured grass carp pituitary cells as model, high-throughput RNA-seq was used to examine the E2-induced differentially expressed genes (DEGs). Transcriptomic analysis showed that E2 could significantly upregulate the expression of 28 genes in grass carp pituitary cells, which were characterized into different functions including reproduction, gonad development, and central nervous system development. Further studies confirmed that E2 could induce luteinizing hormone (LH) and follicle-stimulating hormone (FSH) secretion and mRNA expression in prepubertal grass carp pituitary *in vivo* and *in vitro*. In the pituitary, LH and FSH regulation by E2 were mediated by both ERβ and GPER1. Apparently, E2-induced LHβ and FSHβ mRNA expression were mediated by adenylyl cyclase/cAMP/protein kinase A, phospholipase C/inositol 1,4,5-triphosphate/protein kinase C, and Ca^2+^/calmodulin/CaM-dependent protein kinase II pathways. In addition to LH and FSH, E2 could also induce growth regulation by estrogen in breast cancer 1 (a novel regulator for pituitary development) mRNA expression in grass carp pituitary cells. These results, as a whole, suggested that E2 could play an important role in gonadotropin hormone release and pituitary development in prepubertal grass carp.

## Precis

Investigated the pituitary actions of E2 in prepubertal grass carp by using RNA-seq and demonstrated the receptor specificity and signal transduction for LH and FSH regulation by E2 in grass carp pituitary.

## Introduction

Estrogen, probably the most studied steroid hormone, plays a significant role in vertebrate physiology (1). In mammals, 17β-estradiol (E2) has been proved to play an important role in the hypothalamus–pituitary–gonadal (HPG) axis. The pituitary is the critical center of HPG axis, which receives signals from the hypothalamus and secretes several important hormones including metabolism (TSH), growth (GH), reproduction [luteinizing hormone (LH), follicle-stimulating hormone (FSH)], stress responses (ACTH), lactation (PRL), and other homeostatic functions in multiple organs (2). In mammals, previous studies have reported a direct estrogenic effect on the expression of several pituitary hormones, such as GH (3) and LH (4–7). In teleost, recent studies have also reported that E2 could induce LH secretion and mRNA expression in zebrafish pituitary *in vivo* and *in vitro* (5–7). Similar estrogenic actions were also found in other teleosts, such as croaker (8), Japanese eel (9), and goldfish (10). Except for LH, however, little is known about other E2-regulated genes in teleost pituitary.

Physiological effects of estrogens are mediated by the classical nuclear estrogen receptors [nERs, estrogen receptor alpha (ERα) and ERβ], which belong to the nuclear receptor superfamily members that act as nuclear transcription factors, binding to estrogen response elements within specific genes to alter their rate of transcription (11). Previous studies have reported that high levels of ERα and ERβ were both expressed in human pituitary (12, 13). Meanwhile, pituitary-specific knockout of ERα could cause defects in both positive and negative estrogen feedback regulation of LH in mouse (4). In zebrafish, the three nER isoforms [ERα, estrogen receptor beta 1 (ERβ1), and estrogen receptor beta 2 (ERβ2)] are all detected highly in the pituitary (7). Consistently, recent studies also reported that loss of the ERα and ERβ could lead to an arrest of folliculogenesis at previtellogenic stage II followed by sex reversal from female to male (14). Further studies showed that E2 could bind with ERβ to induce LH secretion and synthesis at the pituitary level in prepubertal zebrafish (5, 6). These studies, as a whole, suggested that ERs played an important role in the teleost pituitary.

In addition to the nERs, it has become clear that estrogens also exert rapid, non-genomic effects by altering different signaling pathways in both central nervous system and peripheral tissues (15). These “non-genomic effects” could mainly be mediated by non-classical membrane bound receptors such as G protein-coupled estrogen receptor (GPER) (16). In mammals, GPER has been identified in the rat brain and pituitary, using immunohistochemistry and *in situ* hybridization (17, 18). In addition, Rudolf and Kadokawa (19) found that GPER was identified in bovine pituitary and might partially contribute to rapid negative estradiol feedback of GnRH-induced LH secretion. In teleost, however, little is known about the functional role of GPER in the pituitary.

To examine the pituitary actions of E2 in grass carp, the cDNAs of grass carp nERs and GPERs were cloned and their expression profile were characterized in brain–pituitary axis. Using primary culture of grass carp pituitary cells as a model, the effects of E2 on pituitary genes expression were examined by high-throughput RNA-seq technique. Then, using real-time PCR and fluorescence immunoassay (FIA), we further examined the direct effects of E2 on pituitary LH, FSH, and growth regulation by estrogen in breast cancer 1 (GREB1) expression in grass carp *in vivo* and *in vitro*. Using various nER and GPER antagonists and agonists, the functional roles of nERs and GPERs in LH, FSH, and GREB1 responses induced by E2 were examined. Finally, using several pharmacological inhibitors, the possible involvement of adenylyl cyclase (AC)/cAMP/protein kinase A (PKA), phospholipase C (PLC)/inositol 1,4,5-triphosphate (IP3)/protein kinase C (PKC), and Ca^2+^/calmodulin (CaM)/CaM-dependent protein kinase II (CaMK-II) pathways in the regulatory actions of E2 on LH and FSH secretion and mRNA expression were also investigated. Our studies further shed light on the pituitary actions and pituitary transduction mechanisms for E2 in fish model.

## Materials and Methods

### Animals and Chemicals

One-year-old grass carp (1+) (*Ctenopharyngodon idellus*) with a body weight (BW) of 1.5–2.0 kg were acquired from local markets and kept in a well-aerated 250-l aquaria at 20 ± 2°C. Since the grass carp at this stage was prepubertal and sexual dimorphism was not apparent, fish of mixed sexes were used for pituitary cell preparation. During this process, the carps were anesthetized in 0.05% MS222 (Sigma, St. Louis, MO, USA) followed by spinosectomy according to the regulations of animal use at the Huazhong Agricultural University (Ethical Approval No. HBAC20091138; Date: November 15, 2009). Seventeen β-estradiol (E2, CAS: 50-28-2) was purchased from Sigma-Aldrich (MO, USA) and resolved in absolute ethanol, and stored frozen at −80°C as 10 mM stocks in small aliquots. Grass carp cGnRH and sGnRH were synthesized by GenScript (Piscataway, NJ, USA). Fulvestrant (ICI182780, CAS: 129453-61-8), propylpyrazole triol (PPT, CAS: 263717-53-9) (5, 6, 20), G-1 (CAS: 881639-98-1), G-15 (CAS: 1161002-05-6) (21), and 2,3-bis(4-hydroxyphenyl) propionitrile (DPN, CAS: 1428-67-7) (22) were purchased from Cayman Chemical (Ann Arbor, MI, USA). These pharmacological agents were prepared as 10 mM frozen stocks in small aliquots and diluted with pre-warmed culture medium to appropriate concentrations 15 min prior to drug treatment.

### Molecular Cloning and Tissue Distribution of Grass Carp nERs and GPERs

Total RNA was extracted from grass carp pituitary, and reverse transcribed into cDNA with SuperScript III (Thermo Fisher Scientific, CA, USA). Full-length ORF region for grass carp ERα, ERβ1, ERβ2, GPER-1a, and GPER-1b were isolated from grass carp pituitary using primers designed based on the sequences in grass carp genomes. Phylogenetic analysis of target sequences based on the corresponding cDNA sequences reported in other species was conducted with MEGA6.0 using neighbor-joining method. Based on the amino acid sequence deduced, three-dimensional protein models for grass carp ERα, ERβ1, ERβ2, GPER-1a, and GPER-1b were constructed using SWISS-MODEL.^1^ For tissue expression, profiling of ERα, ERβ1, ERβ2, GPER-1a, and GPER-1b, reverse transcription PCR (RT-PCR) was conducted in total RNA isolated from various brain areas and pituitary using primers specific for the respective gene targets. In these studies, RT-PCR for β-actin was also performed to serve as an internal control (for primer sequences and PCR condition, please refer to Table S1 in Supplementary Material).

### RNA-Seq and Bioinformatics

Grass carp pituitary cells were prepared by trypsin/DNase II digestion method as described previously (23). After that, pituitary cells were seeded in poly-d-lysin (0.5 mg/ml) precoated 24-well cluster plate at a density of 2.5 × 10^6^ cells/well and incubated for 15–18 h in plating medium with 5% FBS at 28°C under 5% CO_2_ and saturated humidity. After that, culture medium of different wells was, respectively, added with vehicle and 17β-estradiol and the cells were allowed to incubate at 28°C for another 24 h. After drug treatment, total RNA was isolated from individual well using Trizol reagent. The RNA was treated with DNase I to remove contaminating genomic DNA. A Nanodrop 2000 spectrophotometer was used to assess sample purity and RNA concentration, and the quality of the RNA was analyzed on an Agilent 2100 bioanalyzer using the RNA 6000 Nano kit (Agilent Technologies, Santa Clara, CA, USA). RNA (RIN > 8.0) from pituitary cells in control group (three replicates) and E2-treatment group (three replicates) were sent to Majorbio (Shanghai, China) for processing and sequencing. Library preparation and sequencing were performed at the Majorbio Genome Center (Shanghai, China) using a TruSeq™ RNA sample prep Kit on poly(A)-purified RNA, then sequenced on an Illumina HiSeq 2500. We chose a read depth of 600 million 150-bp single end reads. The overall quality of the RNA-seq was sufficient with an average of ~90% of the reads mapping to the grass carp genome. All raw-sequence read data were deposited in NCBI Sequence Read Archive (SRA)^2^ with accession number SRP148383 and the accessed date is 2019. 06. 01.

Clean data were obtained by removing reads containing adapter, poly-*N* and low quality reads from raw data. These high-quality clean reads were mapped to the grass carp genome^3^ using TopHat v2.0. Only reads with a perfect match or one mismatch were further analyzed and annotated based on the reference genome. Gene expression levels were estimated by fragments per kilobase of transcript per million fragments (FPKM) mapped during different samples. Differentially expressed genes (DEGs) were identified using the DESeq R package (1.10.1), which provided statistical routines for determining differential expression in digital gene expression data using a model based on the negative binomial distribution. The *P* values were adjusted using the Benjamini and Hochberg’s approach for controlling the false discovery rate (FDR < 0.01). Gene expressions with fold change (FC) > 1.5 and an adjusted *P* value < 0.05 found by DESeq were assigned as differentially expressed. Gene Ontology (GO) enrichment analysis of the DEGs was implemented by the GOseq R packages based Wallenius non-central hyper-geometric distribution for adjusting gene length bias in DEGs (24).

### Real-Time Quantitative PCR Validation

Grass carp pituitary cells were seeded in poly-d-lysin coated 24-well culture plates at a density of 2.5 million/ml/well. On the following day, drug treatment was initiated by replacing the old medium with testing medium containing the appropriate levels of test substances, and the cells were then allowed to incubate at 28°C for the duration as indicated. After drug treatment, total RNA was extracted from individual well using Trizol and reversely transcribed by PrimeScript RT reagent kit (Takara, Dalian, China). The RT samples were subjected to qPCR using a 7500 real time PCR system (Applied Biosystems, USA) with primers specific for grass carp LHβ, FSHβ, and GREB1, respectively (see Table S1 in Supplementary Material for primer sequences and PCR condition). In these experiments, serial dilutions of plasmid DNA with the coding sequences for grass carp LHβ, FSHβ, and GREB1 were used as the standards for data calibration, and parallel real-time PCR for β-actin was also conducted to serve as the internal control.

### FIA for LH

To examine the direct effect of E2 on LH secretion in carp pituitary, a primary culture of pituitary cells was prepared from 15 to 18 prepubertal grass carp by trypsin/DNase II digestion method (23), seeded in 24-well plates at 2.5 million cells per well, and incubated with test substances for the duration as indicated. After that, culture medium was harvested for measurement of hormone release using FIA. For grass carp LH, biotinylated carp LH was prepared and used as the tracer for the respective assays. A 50-µl volume of protein sample was added into individual wells of Costar 96-well black plate (Thermo Fisher Scientific, MA, USA) precoated with protein A (0.5 µg/ml) with 100 µl of reading buffer containing 12.5 ng/ml biotinylated carp LH and 1:10K carp LH antibody (for information of LH antibody, please refer to Table S2 in Supplementary Material). After overnight incubation at 4°C, individual wells were rinsed three times with washing buffer to remove non-specific binding of primary antibody. HRP-conjugate streptavidin (0.5 µg/ml) was then introduced and incubated for another 1 h at room temperature. After that, unbound second antibody was removed by decanting and a 100-µl volume of QuantaBlu™ Fluorogenic Peroxidase Substrate (Thermo Fisher Scientific) was then added into individual wells for signal development. Fluorescence signals were routinely detected using a FluoStar OPTIMA-Fluorecence plate reader (BMG Labtech GmbH, Ortenberg, Germany). The intra- and inter-assay coefficients of variation (the level of ED_50_ of individual assays) are found to be 6.2% (*n* = 12) and 738% (*n* = 12) for LH assay, respectively. The minimal detection limit and ED_50_ value were found to be 0.1 and 1.2 ± 0.09 nM (*n* = 4), respectively. Based on our validation, the FIA assays for LH did not cross-react with other pituitary hormones in grass carp, including GH, FSH, and PRL.

### Western Blot for FSH

Grass carp pituitary cells were seeded in poly-d-lysine coated 24-well culture plates at a density of 2.5 × 10^6^ cells/ml/well and incubated with drug treatment for the duration as indicated in individual experiments. After drug treatment, culture medium from individual well was removed, and remaining cells were rinsed with PBS and lysed in RIPA buffer (50 mM Tris–HCl, 150 mM NaCl, 1 mM EDTA, 1% NP-40, and 0.25% Na deoxycholate) containing a final concentration of 1× protease inhibitor cocktail (Roche). The cells lysate was cleared by high-speed centrifugation at 4°C, and the clear supernatant was resolved in 10% gel by SDS-PAGE. The antibody for grass carp FSH (1:5,000) was used at the dilutions recommended by our validation. Following the incubation, the membrane was washed three times to remove non-specific binding of primary antibodies and the HRP-conjugated secondary antibodies [goat anti-rabbit IgG (1:5,000)] were introduced for signal development. Chemiluminescence signals for target immune-reactivity were detected using SuperSignal West Pico (PIERCE, Rockford) as the substrate and quantified using the IC440 CF Digital Science Image Station (Eastman Kodak). In these experiments, the pixel density of the western blot bands were quantified with ImageJ^4^ and the Western blot of β-actin was used as an internal control using its antibody (1:15,000) (for information of β-actin and FSH antibody, please refer to Table S2 in Supplementary Material).

### *In Vivo* Estradiol Treatments and Sampling Procedure

After entraining the grass carp in 250-l tanks with the one-meal-per-day feeding schedule, drug treatment by intraperitoneal (IP) injection was performed as described previously (25). Twenty-four prepubertal grass carps (BW: 850 ± 75 g) were divided into two experimental groups (*n* = 12 carps/group). Each grass carp received one intraperitoneal injection of 2 ng E2/g BW suspended in 0.15 M NaCl or vehicle alone (control). After treatment, the blood samples from each fish were collected in 3, 6, and 24 h, respectively, by using the vacuum blood collection tube. After 24 h, the pituitary was collected from each fish and stored in liquid nitrogen until the mRNA extraction.

### Data Transformation and Statistical Analysis

For LH FIAs, standard curves with a range from 0.98 to 500 ng/ml and ED_50_ value of 8–15 ng/ml were used for data calibration with a four-parameter logistic equation of the GraphPad Prism program (GraphPad, San Diego, CA, USA). For real-time PCR of LHβ, FSHβ and GREB1 mRNA, standard curves with dynamic range of 10^5^ and correlation coefficient >0.95 were used for data calibration with ABI7500 software. Since no significant changes were noted for β-actin mRNA in our studies, LHβ, FSHβ, and GREB1 mRNA data as well as LH protein data were simply transformed as a percentage of the mean value in the control group without drug treatment (as “% Ctrl”). The data presented (as mean ± SEM) were pooled results from 6 to 8 experiments and analyzed with ANOVA followed by Dunnett’s test using Prism 6.0 and differences between groups were considered as significant at *P* < 0.05.

## Results

### Molecular Cloning and Sequence Analysis of Estrogen Receptor in Grass Carp Pituitary

**Figure 1:**
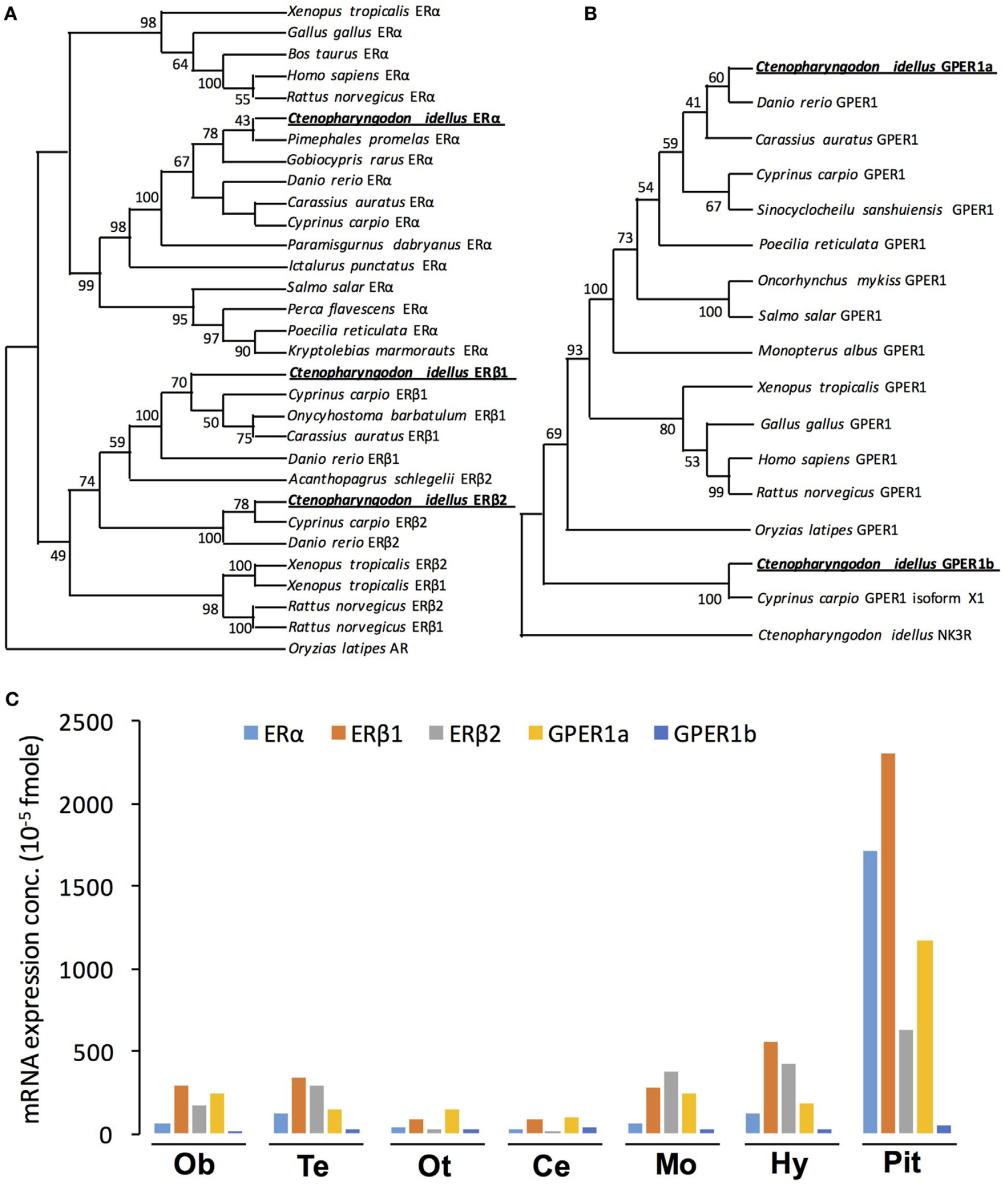
Phylogenetic analysis and tissue distribution of ERs and protein-coupled estrogen receptors (GPERs) in grass carp. **(A)** Phylogenetic analysis of ER amino acid sequences in vertebrate species using neighbor-joining method with MEGA6.0. The *Oryzias latipes* AR was used as the outgroup. **(B)** Phylogenetic tree of grass carp GPER using predicted amino acid sequences using neighbor-joining method with MEGA6.0 program. NK3R from grass carp served as an outgroup to root the tree. **(C)** Tissue distribution of estrogen receptor alpha (ERα), estrogen receptor beta 1 (ERβ1), estrogen receptor beta 2 (ERβ2), GPER1a, and GPER1b in grass carp brain areas. Total RNA was isolated from brain areas in grass carp and subjected to reverse transcription PCR (RT-PCR) using primers specific for carp ERα, ERβ1, ERβ2, GPER1a, and GPER1b transcripts, respectively. Parallel RT-PCR for β-actin was also conducted to serve as the internal control.

In this study, three nERs were isolated from grass carp pituitary, namely, ERα (GenBank No.: MG696766), ERβ1 (GenBank No.: MG735677), and ERβ2 (GenBank No.: MG645389), respectively (Figure S1 in Supplementary Material). The whole amino acid sequence of ERα was found to share 43.0 and 44.9% identity with that of ERβ1 and ERβ2 in grass carp, respectively (Figure S2A in Supplementary Material). The ERβ1 and ERβ2 shared 56.7% identity in their whole amino acid sequences (Figure S2A in Supplementary Material). Phylogenetic analysis using neighbor-joining method based on the amino acid sequences of nERs reported in vertebrates reveals that the newly cloned grass carp ERα, ERβ1, and ERβ2 can be clustered in the clade of fish ERα, ERβ1, and ERβ2, respectively (Figure 1A). Grass carp ERα showed a close evolutionary relationship with *Pimephales promelas* ERα (Figure 1A). Within the clade of ERβ, grass carp ERβ1 and ERβ2 displayed a closer evolutionary relationship with common carp ERβ1 and ERβ2, respectively (Figure 1A).

In addition to the three nERs, two membrane receptors, named GPER1a (GenBank No.: MG696765) and GPER1b (GenBank No.: MG770453), were also successfully isolated from grass carp pituitary (Figure S1 in Supplementary Material). *In silico* protein modeling using SWISS-MODEL program confirmed that both GPER1a and GPER1b were seven-transmembrane domain G protein-coupled receptors (Figure S2D in Supplementary Material). Sequence alignment showed that the GPER1a and GPER1b shared 52.5% identity in their whole amino acid sequences (Figure S2C in Supplementary Material). Phylogenetic analysis based on the amino acid sequence further confirmed that the newly cloned GPER1a and GPER1b could be both clustered in the clade of GPER family (Figure 1B). Similar to other vertebrates, grass carp GPER1a had a close evolutionary relationship with zebrafish GPER1, while grass carp GPER1b was only clustered with common carp GPER1 isoform x1.

### Tissue Distribution of nERs and GPERs in Grass Carp

To establish the brain expression profile of five estrogen receptors (GPER1a, GPER1b, ERα, ERβ1, and ERβ2), RT-PCR was performed in various brain areas. As shown in Figure 1C, transcript signals for the five receptors (GPER1a, GPER1b, ERα, ERβ1, and ERβ2) were all detected widely in olfactory bulb, telencephalon, optic tectum, cerebellum, medulla oblongata, hypothalamus, and pituitary. The highest expression levels of GPER1a, ERα, ERβ1, and ERβ2 were all detected in the pituitary (Figure 1C). In the pituitary, the transcript signals for ERβ1 were higher than other estrogen receptors. In addition, GPER1b displayed the lowest expression level in the pituitary compared with the other four estrogen receptors.

### RNA-Seq Analysis

To investigate the direct estrogenic effect in teleost pituitary, E2 (100 nM) was used to incubate the primary cultured pituitary cells derived from prepubertal grass carp. After 24 h challenge, a high-throughput RNA-seq technique was used to compare mRNA expression profiles between control and E2-treatment group. In this study, the cDNA libraries from E2-treatment group and control group were established and sequenced. The RNA-Seq of six samples yielded around 599.6 million raw paired-end reads. After quality filtering, each sample remained approximately 52.5 million high-quality clean reads. Based on the fragments per kilobase of transcript per million fragments (FPKM) value, total 26,224 unigenes were identified in grass carp pituitary, while 96.4% of these unigenes were expressed at low abundance (FPKM value <100). Most of the hormone precursor genes were the highest expression unigenes in the pituitary, such as GH, POMC, prolactin, somatolactin (SLα and SLβ), LH, and FSH. FPKM analysis showed that 64 unigenes (FC > 1.5 and FDR < 0.05) were differentially expressed after E2 treatment, among which 28 genes were upregulated and 36 genes were downregulated (Figure S3 in Supplementary Material). The top 10 upregulated genes were as follows: DERL3, PLP1, GREB1, LHβ, HAPLN3, PNGase F, PLAU, FBN1, NR5A1, and APOC, respectively (Table 1). The top 10 downregulated genes were as follows: RYR3, RFX1, GRN, SGSM1, TBX20, CELSR1, FAM13A, ABCA3, SFRP2, and ATP2B, respectively (Table 2). GO analysis for the DEGs revealed that most upregulated DEGs were involved in reproduction, central nervous system development, gonad development, protein folding, and calcium ion homeostasis (Table 1). However, 36 downregulated DEGs were involved in calcium ion binding, steroid process, GTPase activator activity, Wnt signaling pathway, IGF binding, heparin binding, ATPase binding and activity, DNA/RNA binding, and metalloendopeptidase inhibitor activity (Table 2).

**Table 1:** Genes upregulated by E2 in prepubertal grass carp pituitary cells.

Gene	Description	Fold change	GO-biological process
*DERL3*	Derlin-3	10.21	Degradate misfolded glycoproteins
*PNGase F*	Peptide-*N*4-*N*-acetyl-beta-d-glucosaminyl asparagine amidase F	3.86	Degradate misfolded glycoproteins
*LHβ*	Luteinizing hormone beta subunit	5.19	Reproduction
*FSHβ*	Follicle-stimulating hormone beta subunit	1.82	Reproduction
*DRD2*	Dopamine D2 receptor (GPCR)	1.79	Reproduction
*GREB1*	Growth regulation by estrogen in breast cancer 1	5.78	Estrogen receptor binding
*RNF4*	E3 ubiquitin-protein ligase RNF4	1.73	Estrogen receptor binding
*PGR*	Progesterone receptor 1	1.71	Steroid binding
*APOC*	Apolipoprotein C Ia	2.25	Cholesterol metabolic process
*NUPR1*	Nuclear protein 1	2.23	Male gonad development
*NR5A1*	Nuclear receptor subfamily 5 group A (SF-1)	2.34	Male gonad development
*DRC7*	Dynein regulatory complex subunit 7	1.68	Spermatogenesis
*PLP1*	Proteolipid protein 1b	6.3	Central nervous system development
*HAPLN3*	Hyaluronan and proteoglycan link protein 3	4.43	Central nervous system development
*NRP1*	Neuropilin-1a isoform 1 precursor	1.83	Central nervous system development
*S100B*	S100 calcium binding protein, beta (neural)	2.1	Central nervous system development
*LRRC4*	Leucine-rich repeat-containing protein 4	2.08	Synaptic adhesion protein
*SYT13*	Synaptotagmin-13 precursor	2.19	Synaptic vesicle transport
*OCLN*	Occludin-like	2.03	Cell–cell junction organization
*FBN1*	Fibrillin-1-like	2.61	Cell proliferation
*PLAU*	Plasminogen activator PLAU	3.19	Cell proliferation
*FGFR4*	Fibroblast growth factor receptor 4	1.63	Cell proliferation
*HYAL2*	Hyaluronidase-2-like	1.71	Cellular response to FGF stimulus
*PPT2*	Lysosomal thioesterase PPT2-like	1.61	Fatty-acyl-CoA biosynthetic process
*TRDMT1*	tRNA (cytosine-5-)-methyltransferase	1.77	Methyltransferase
*TMEM53*	Transmembrane protein 53	1.81	Integral component of membrane
*FAM222A*	Family with sequence similarity 222, member A	1.73	Protein dimerization activity
*TMTC2*	Transmembrane and TPR repeat-containing protein 2	1.67	Calcium ion homeostasis

**Table 2:** Genes downregulated by E2 in prepubertal grass carp pituitary cells.

Name	Description	Fold change	GO-molecular function
*RYR3*	Ryanodine receptor 3	0.39	Calcium ion binding
*ATP2B*	Membrane calcium-transporting ATPase 2	0.56	Calcium-transporting
*CELSR1*	Cadherin EGF LAG seven-pass G-type receptor 1	0.54	Calcium ion binding
*LDLR*	Low-density lipoprotein receptor-like	0.60	Calcium ion binding
*GRN*	Granulin	0.50	Response to estradiol
*DHCR24*	24-dehydrocholesterol reductase	0.60	Steroid metabolic process
*LSS*	Lanosterol synthase	0.60	Steroid biosynthetic process
*NSDHL*	Sterol-4-alpha-carboxylate 3-dehydrogenase	0.59	Steroid dehydrogenase activity
*SCRIB*	Protein scribble homolog isoform X8	0.59	GTPase activator activity
*SGSM1*	Small G protein signaling modulator 1	0.52	GTPase activator activity
*FAM13A*	Protein FAM13A isoform X2	0.55	GTPase activator activity
*SFRP2*	Secreted frizzled-related protein 2	0.56	Wnt-protein binding
*CTNNB1*	Catenin beta-1-like	0.56	Wnt signaling pathway
*CD2AP*	CD2-associated protein	0.57	Beta-catenin binding
*ZNFX1*	NFX1-type zinc finger-containing protein 1-like	0.59	Transcription factor activity
*TSC22D3*	TSC22 domain family protein 3	0.59	Transcription factor activity
*TIMP3*	Metalloproteinase inhibitor 3-like	0.58	Metalloendopeptidase inhibitor
*TIMP2*	Tissue inhibitor of metalloproteinase 2	0.59	Metalloendopeptidase inhibitor
*BRD4*	Bromodomain-containing protein 4	0.59	NF-kappa B signaling
*JIP2*	c-Jun-amino-terminal kinase-interacting protein	0.58	JNK/MAPK cascade
*PHIP*	PH-interacting protein isoform X2	0.60	Insulin receptor binding
*CRIM1*	Cysteine rich transmembrane BMP regulator 1	0.58	IGF binding
*IGF1R*	IGF-I receptor subtype a	0.58	IGF binding
*COL5AS*	Collagen alpha-1 (V) chain	0.59	Heparin/integrin binding
*PTPRF*	Receptor-type tyrosine-protein phosphatase F	0.58	Heparin binding
*NAV2*	Neuron navigator 2 isoform X1	0.58	Heparin binding
*ANK*	Ankyrin-2-like	0.59	ATPase binding
*ABCA3*	ATP-binding cassette sub-family A member 3	0.55	ATPase activity
*RAPGEF2*	Rap guanine nucleotide exchange factor 2	0.60	Adrenergic receptor binding
*FN1*	Fn1 protein	0.56	Adrenal gland development
*TBX20*	T-box transcription factor TBX20	0.53	DNA binding
*RFX1*	MHC class II regulatory factor RFX1	0.43	DNA binding
*ADARB*	Adenosine deaminase, RNA-specific, B1	0.58	RNA binding
*FAM120C*	Family with sequence similarity 120C	0.60	RNA binding
*SSTR3*	Somatostatin receptor type 3	0.60	Neuropeptide binding
*MAP1*	Microtubule-associated protein 1A-like	0.59	Ubiquitin protein ligase binding

### Pituitary Gene Regulation by E2

**Figure 2:**
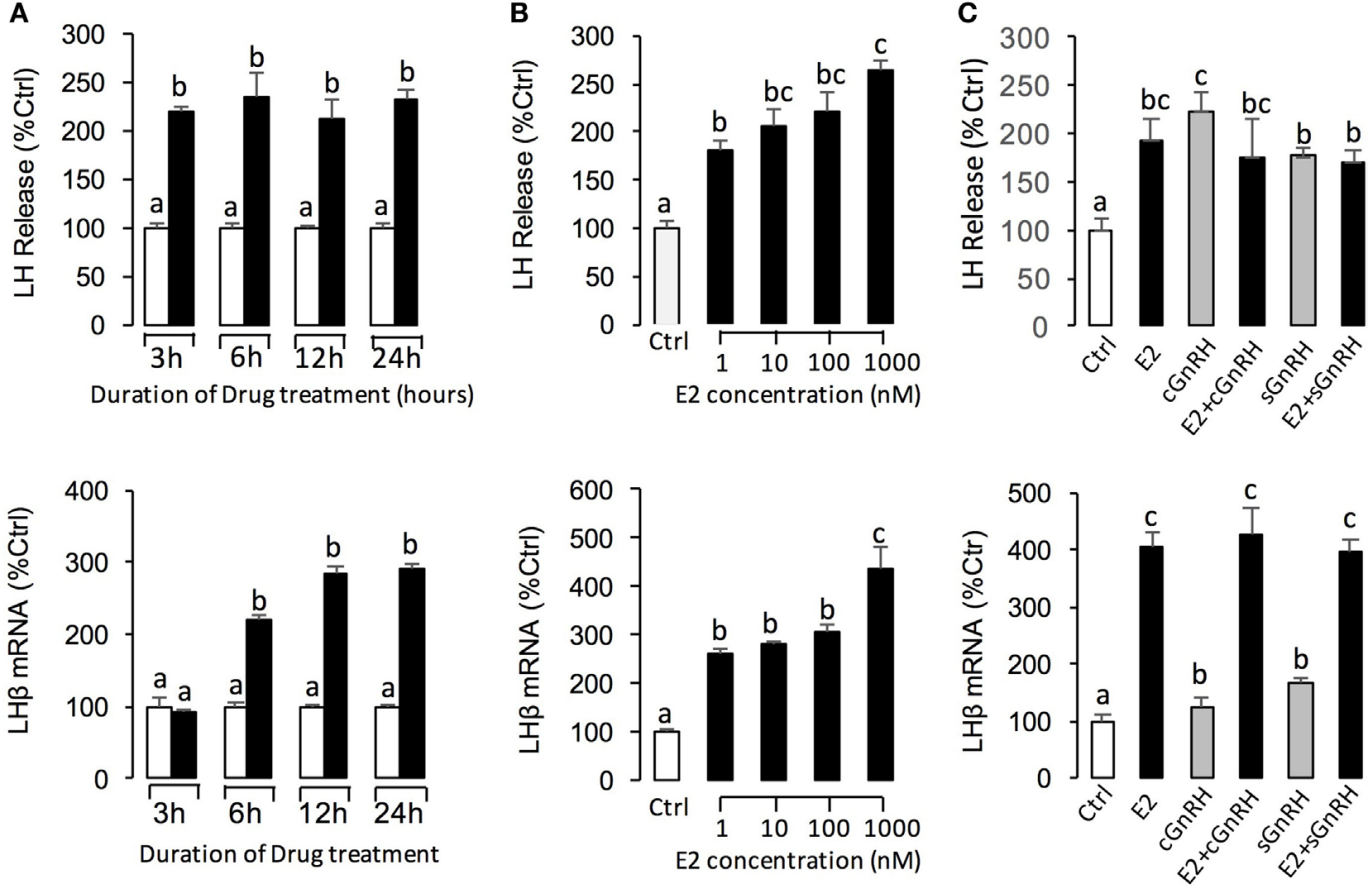
E2 induce luteinizing hormone (LH) secretion and mRNA expression in grass carp pituitary cells. **(A)** In the time-course experiment, pituitary cells were incubated with E2 (10 nM) for the duration as indicated. **(B)** In parallel studies for dose dependence, pituitary cells were challenged for 24 h with increasing levels of E2 (1–1,000 nM). **(C)** Effects of E2 (10 nM), sGnRH (1 µM), and cGnRH (1 µM) on LH and follicle-stimulating hormone (FSH) mRNA expression in grass carp pituitary cells. After drug treatment, culture medium was harvested for measurement of LH secretion, and the remaining cells were used for total RNA preparation for real-time PCR of the LHβ and FSHβ mRNA. Data presented are expressed as mean ± SEM, and groups denoted by different letters represent a significant difference at *P* < 0.05 (ANOVA followed by a Dunnett’s test).

**Figure 3:**
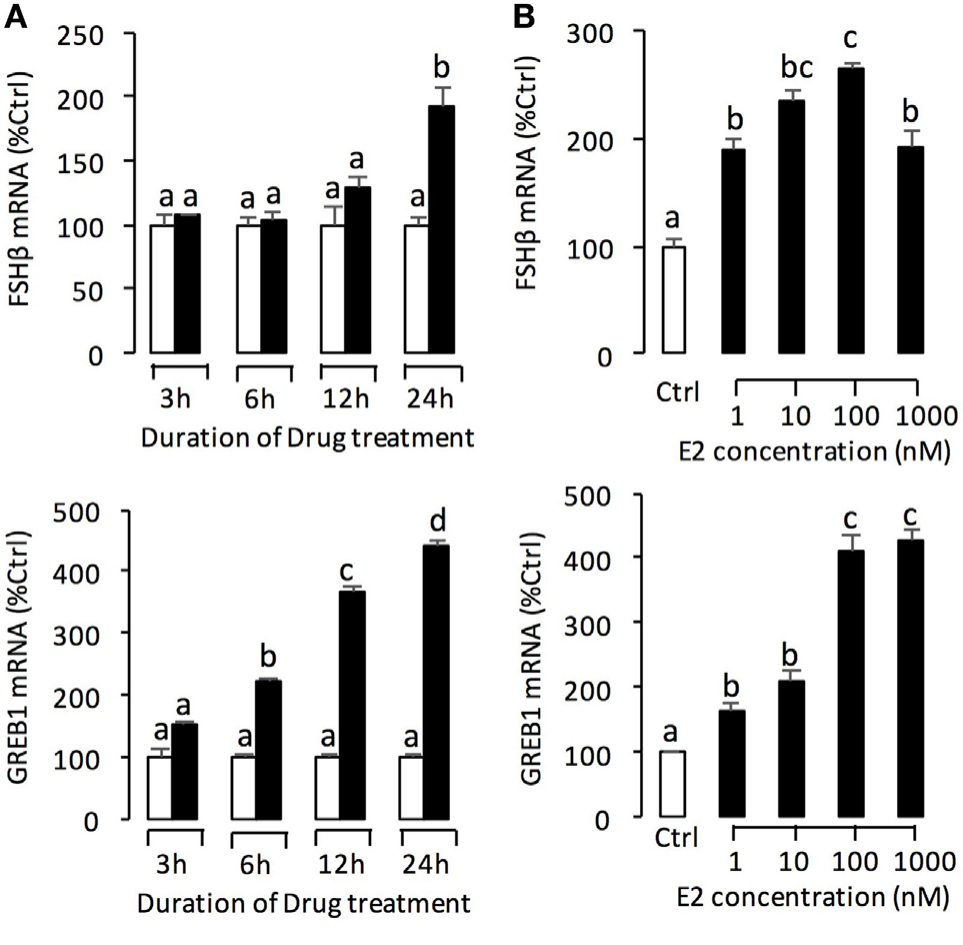
E2 induce follicle-stimulating hormone (FSH)β and growth regulation by estrogen in breast cancer 1 (GREB1) mRNA expression in grass carp pituitary cells. **(A)** In the time-course experiment, pituitary cells were incubated with E2 (100 nM) for the duration as indicated. **(B)** In parallel studies for dose dependence, pituitary cells were challenged for 24 h with increasing levels of E2 (1–1,000 nM). After drug treatment, the remaining cells were used for total RNA preparation for real-time PCR of the FSHβ and GREB1 mRNA expression. Data presented are expressed as mean ± SEM, and groups denoted by different letters represent a significant difference at *P* < 0.05 (ANOVA followed by a Dunnett’s test).

To further confirm the pituitary actions of E2, primary culture of grass carp pituitary cells were incubated by E2 again. Time-course experiments revealed that E2 could trigger LH secretion and LHβ mRNA expression up to 3 and 6 h, respectively (Figure 2A). In dose-dependent studies, 24-h incubation with increasing concentrations of E2 (1–1,000 nM) could induce LH secretion and mRNA expression in a dose-dependent fashion (Figure 2B). In addition to LH, time-course experiment also revealed that E2 could significantly induce GREB1 mRNA expression up to 3 h and induce FSHβ mRNA expression up to 12 h with maximal effect at 24 h (Figure 3A). In dose-dependent studies, 24-h incubation with increasing concentrations of E2 (1–1,000 nM) could also triggered GREB1 and FSHβ mRNA expression in a dose-related fashion (Figure 3B). To compare the functional role of E2 and GnRH on LH secretion and mRNA expression in the carp pituitary, co-treatment of E2 with sGnRH or cGnRH was performed in the carp pituitary cells. As shown in Figure 2C, static incubation with E2 (10 nM), sGnRH (1 µM), and cGnRH (1 µM) alone were all effective in increasing LH secretion and mRNA expression in carp pituitary cells. Compared to sGnRH or cGnRH, E2 was more effective in inducing pituitary LHβ mRNA expression. In addition, the stimulatory effects on LH secretion and mRNA expression were not markedly enhanced with simultaneous exposure to E2 with sGnRH or E2 with cGnRH (Figure 2C).

### Receptor Specificity of E2-Induced Gene Expression in Grass Carp Pituitary Cells

**Figure 4:**
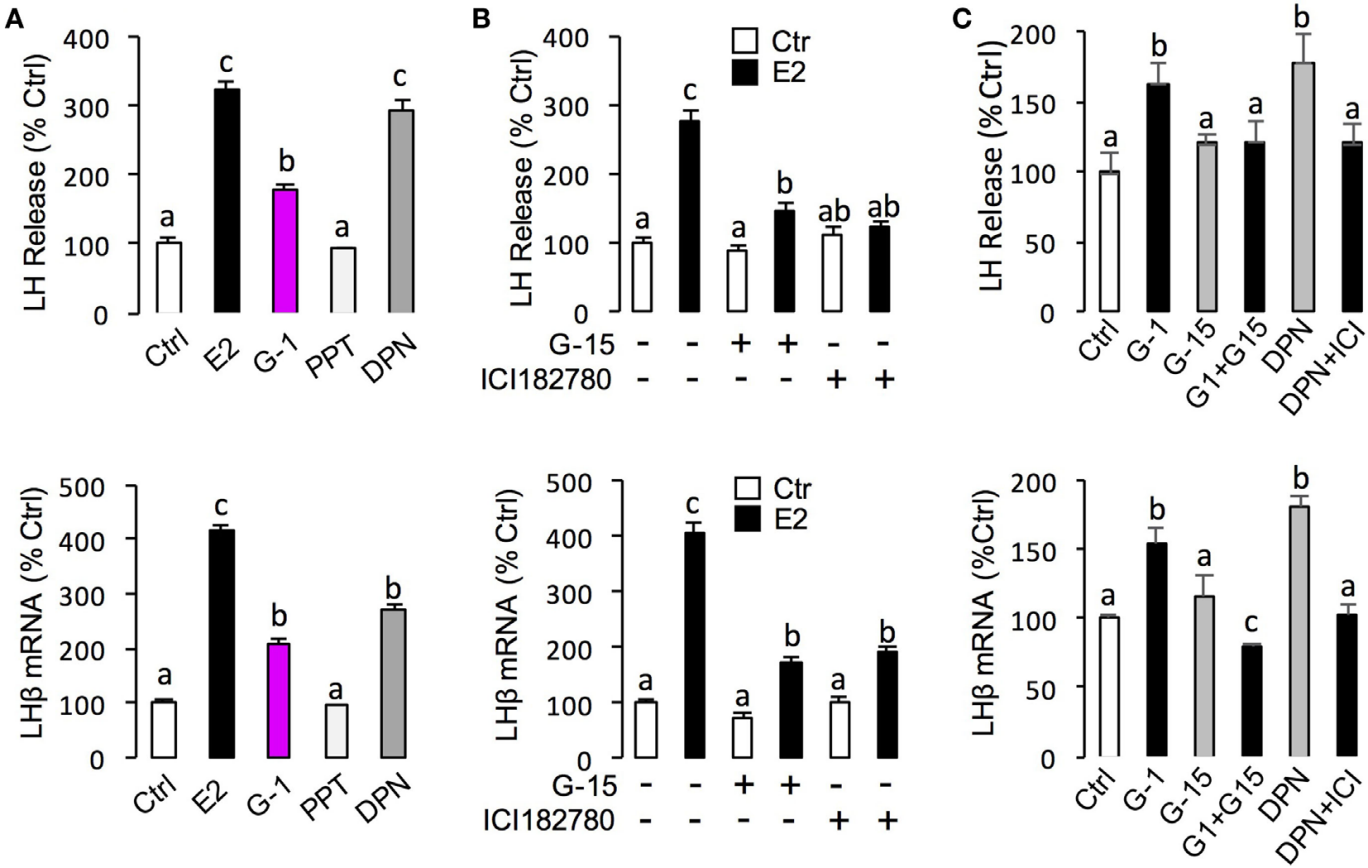
Receptor specificity of E2-induced luteinizing hormone (LH) secretion and mRNA expression. **(A)** Effects of ER agonists on LH transcript expression and hormone secretion. For LHβ mRNA expression, pituitary cells were treated for 24 h with E2 (10 nM), G protein-coupled estrogen receptor (GPER) agonist G-1 (1 µM), estrogen receptor alpha agonist propylpyrazole triol (PPT) (1 µM), and ERβ agonist DPN (1 µM), respectively. **(B)** Effects of GPER antagonist G-15 and ER antagonist ICI182780 on E2-induced LHβ secretion and mRNA expression. Pituitary cells were treated for 24 h with E2 (10 nM) in the presence or absence of the G-15 (10 µM) or ICI182780 (10 µM), respectively. **(C)** Effects of GPER antagonist G-15 or ER antagonist ICI182780 on G-1- or DPN-induced LH secretion and mRNA expression. Grass carp pituitary cells were incubated for 24 h with GPER agonist G1 (1 µM) in the presence or absence of G-15 (10 µM), or ERβ agonist DPN (1 µM) in the presence or absence of ICI182780 (10 µM).

In this experiment, a pharmacological approach was recruited to clarify the receptor specificity for the pituitary actions of E2 in grass carp. In this case, pituitary cells were incubated for 24 h with E2 (10 nM) in the presence of the ER antagonist ICI182,780 (10 µM) or GPER antagonist G-15 (10 µM), respectively. As shown in Figure 4B, the stimulatory effects of E2 on LH release and mRNA expression could be blocked by co-treatment with ICI182,780 or G-15. Consistent with these results, the E2-induced LH secretion and LHβ mRNA expression could be mimicked by ERβ agonist DPN (1 µM) and GPER agonist G-1 (1 µM) but not ERα agonist PPT (1 µM) (Figure 4A). In addition, G-1 (1 µM)- and DPN (1 µM)-induced LH secretion and mRNA expression could be totally blocked by co-treatment with G-15 (10 µM) or ICI182780 (10 µM), respectively (Figure 4C).

**Figure 5:**
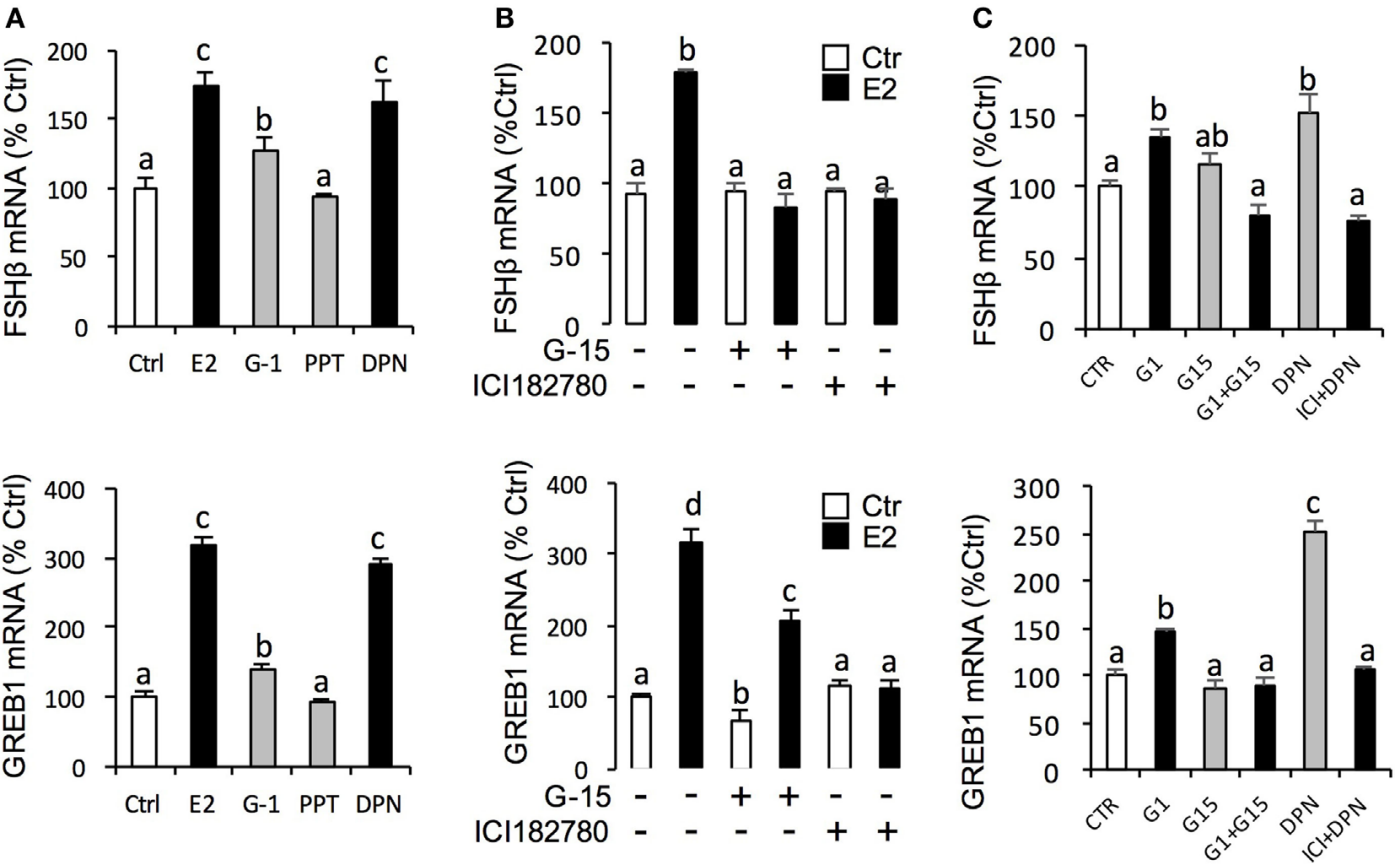
Receptor specificity of E2-induced follicle-stimulating hormone (FSH)β and growth regulation by estrogen in breast cancer 1 (GREB1) mRNA expression. **(A)** Effects of ER agonists on FSHβ and GREB1 mRNA expression. Pituitary cells were treated for 24 h with E2 (10 nM), G protein-coupled estrogen receptor (GPER) agonist G-1 (1 µM), estrogen receptor alpha agonist propylpyrazole triol (PPT) (1 µM), and ERβ agonist DPN (1 µM), respectively. **(B)** Effects of GPER antagonist G-15 and ER antagonist ICI182780 on E2-induced FSHβ and GREB1 mRNA expression. Pituitary cells were treated for 24 h with E2 (10 nM) in the presence or absence of the G-15 (10 µM) or ICI182780 (10 µM), respectively. **(C)** Effects of GPER antagonist G-15 or ER antagonist ICI182780 on G-1- or DPN-induced FSHβ or GREB1 mRNA expression. Grass carp pituitary cells were incubated for 24 h with GPER agonist G1 (1 µM) in the presence or absence of G-15 (10 µM), or ERβ agonist DPN (1 µM) in the presence or absence of ICI182780 (10 µM).

**Figure 6:**
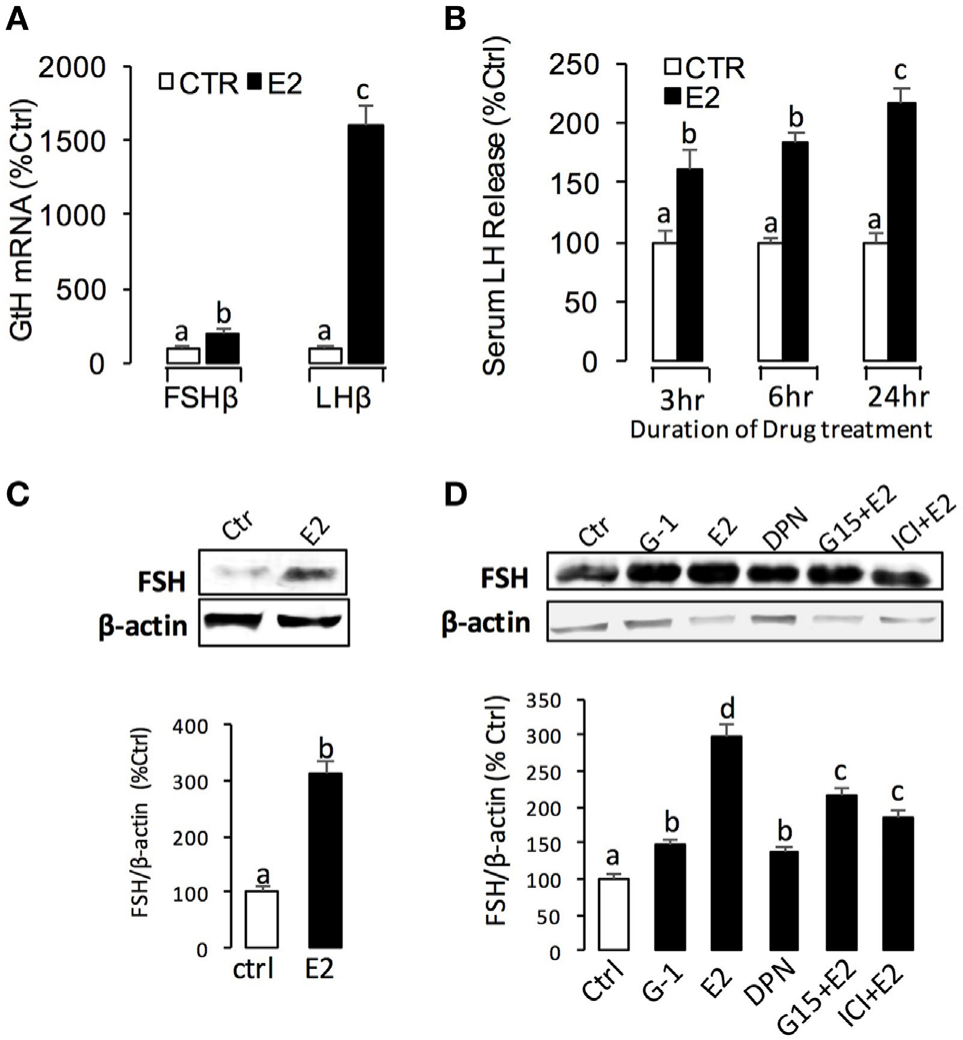
E2-induced luteinizing hormone (LH) and follicle-stimulating hormone (FSH) secretion and mRNA expression in grass carp. **(A)** *In vivo* effects of E2 on LHβ and FSHβ mRNA expression in grass carp pituitary. **(B)** E2-induced serum LH secretion in prepuberty grass carp. **(C)** Effect of E2 on FSH secretion in the serum of prepuberty grass carp. The quantified graph is shown below the representative blots in each group. Data presented are expressed as mean ± SEM (*n* = 3). **(D)** Receptor specificity of E2-induced FSH synthesis in grass carp pituitary cells. The quantified graph is shown below the representative blots in each group. Data presented are expressed as mean ± SEM (*n* = 3).

For the receptor specificity of E2-induced FSHβ and GREB1 mRNA expression, pituitary cells were treated with E2 (10 nM) in the presence of ER antagonist ICI182,780 (10 µM) or GPER antagonist G-15 (10 µM), respectively. Similar to LH, E2 was effective in stimulating FSHβ and GREB1 mRNA expression, which could be mimicked by the ERβ agonist DPN (1 µM) and GPER agonist G-1 (1 µM) but not ERα agonist PPT (1 µM) (Figure 5A). Furthermore, the corresponding responses for FSHβ and GREB1 mRNA expression could be negated by co-treatment with the GPER antagonist G15 (10 µM) or ER antagonist ICI182,780 (10 µM), respectively (Figure 5B). Finally, G-1(1 µM)- and DPN (1 µM)-induced FSHβ and GREB1 mRNA expression could be totally abolished by co-treatment with G-15 (10 µM) or ICI182780 (10 µM), respectively (**Figure 5C**). In the parallel experiments, E2, G-1, and DPN could all induce FSH protein synthesis in grass carp pituitary cells (Figure 6D). In addition, E2-induced FSH synthesis could be partially blocked by the GPER antagonist G15 or ER antagonist ICI182,780, respectively (Figure 6D).

### Signal Transduction for E2-Induced LHβ and FSHβ mRNA Expression in Grass Carp Pituitary Cells

**Figure 7:**
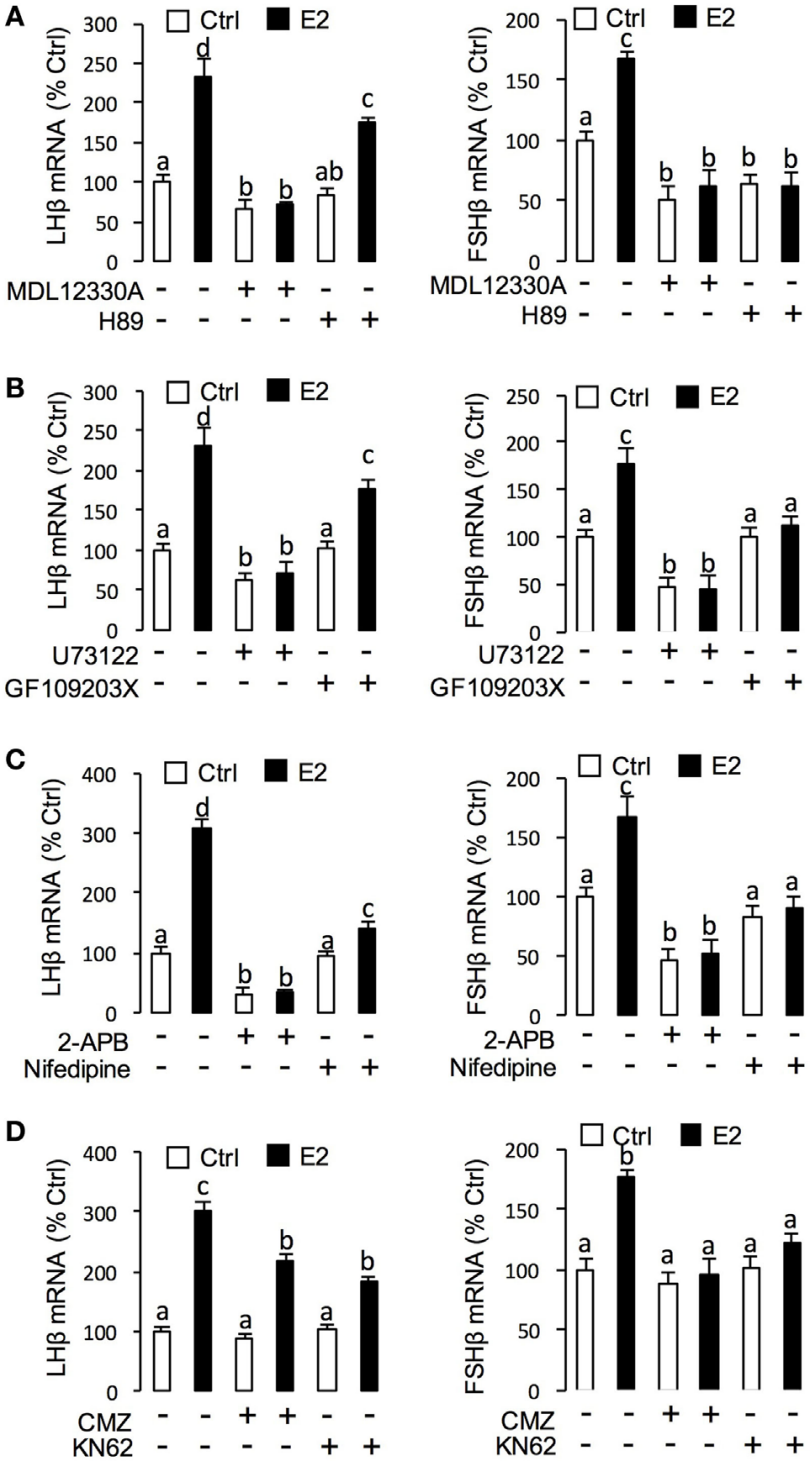
Signal transduction of E2-induced luteinizing hormone (LH)β and follicle-stimulating hormone (FSH)β mRNA expression in grass carp pituitary cells. **(A)** Effects of 24-h co-treatment with the adenylyl cyclase inhibitor MDL12330A (20 µM) or protein kinase A inhibitor (20 µM) on E2 (10 nM)-induced LHβ and FSHβ mRNA expression. **(B)** Effects of 24-h co-treatment with the phospholipase C inhibitor U73122 (10 µM) or protein kinase C inhibitor GF109203X (20 µM) on E2 (10 nM)-induced LHβ and FSHβ mRNA expression in carp pituitary cells. **(C)** Effects of 24-h co-treatment with inositol 1,4,5-triphosphate receptor blocker 2-APB (100 µM) or VSCC blocker Nifedipine (10 µM) on E2 (10 nM)-induced LHβ and FSHβ mRNA expression. **(D)** Effects of 24-h co-treatment with calmodulin antagonist calmidazolium (1 µM) or CaM-dependent protein kinase II inactivator KN62 (10 µM), respectively, on E2 (10 nM)-induced LHβ and FSHβ mRNA expression in grass carp pituitary cells.

To unveil the signal transduction for E2-induced LHβ and FSHβ mRNA expression, the possible involvement of cAMP-dependent cascade was examined using the inhibitors for cAMP pathway. As shown in Figure 7A, co-treatment with AC inhibitor MDL12330A (20 µM) or PKA inhibitor H89 (20 µM) were both effective in blocking the stimulatory effects of E2 on LHβ and FSHβ mRNA expression. In parallel experiments, E2-induced LHβ and FSHβ mRNA expression could be abrogated by simultaneous treatment with the PLC inhibitor U73122 (10 µM), PKC inhibitor GF109203X (10 µM) (Figure 7B), and IP3 receptor blocker 2-APB (100 µM) (Figure 7C). In addition, E2-induced LHβ and FSHβ mRNA expression were also found to be suppressed by co-treatment with the VSCC inhibitor nifedipine (10 µM) (Figure 7C) (26), CaM antagonist calmidazolium (1 µM), or CaMK-II blocker KN62 (10 µM) (Figure 7D), respectively.

### E2 Effect *In Vivo*

Using prepubertal grass carp as model, we tested the biological function of E2 *in vivo*. Single intraperitoneal injection of E2 (2 ng/g BW) could significantly induce LHβ and FSHβ mRNA expression in prepubertal grass carp pituitary after 24 h treatment (Figure 6A). In parallel experiments, E2 could induce serum LH secretion from 3 to 24 h (Figure 6B). Similarly, E2 could also induce serum FSH secretion after 24 h treatment (Figure 6C).

## Discussion

In mammals, estrogens are essential female sex steroids that control all aspects of female development and reproduction (27, 28). Recently, E2 is also known to be involved in zebrafish reproductive regulation and puberty onset through activating its nERs (14); however, little is known regarding its direct effects in fish pituitary. In this study, using primary cultured grass carp pituitary cells as model, we demonstrated the pituitary actions of E2 in teleost.

Estrogens could activate both nuclear and membrane receptors, while most reported effects of estrogens were mediated via the nERs (29, 30). Similar to zebrafish (5, 6), three nER isoforms were also isolated from grass carp, namely, ERα, ERβ1, and ERβ2, respectively. Furthermore, the three nER isoforms were all highly detected in grass carp pituitary. In mammals, the high expression of nERs in the pituitary has also been reported in rats (31), sheep (32), and human (33). These results further confirmed that estrogen responsiveness in the pituitary required the presence of nERs, including the classical ERα and ERβ (34). In addition to the nERs, two GPER isoforms (namely, GPER1a and GPER1b) were also identified in grass carp. The GPERs were also found to be highly expressed in grass carp pituitary, which indicated that GPER might play a role in mediating the non-genomic effects of estradiol in grass carp pituitary.

Recently, transcriptomic analysis has been used to determine the function of E2 in mouse brain (35), arcuate nucleus (36), and cultured human myometrial smooth muscle cells (37), while there was no study to examine the pituitary action of E2 at transcriptomic level. In this study, using high-throughput RNA-seq approach, we found that E2 could significantly stimulate 28 gene expressions in primary cultured grass carp pituitary cells. GO analysis showed that these upregulated DEGs were mainly grouped into reproduction, gonad development, central nervous system development, cell proliferation, steroid receptor binding, and calcium regulation. Previous studies have demonstrated that estrogens could regulate numerous functions in the brain and pituitary including reproduction (38) and neuronal synaptic plasticity (39). These results, as a whole, indicated that E2 could not only directly induce pituitary development but also could stimulate several genes expression in reproductive hormones and other gonad development factors to promote gonad development in prepubertal grass carp. Furthermore, E2 could downregulate 36 transcripts involved in calcium ion binding, GTPase activator activity, Wnt signaling pathway, IGF binding, heparin binding, ATPase binding and activity, DNA/RNA binding, and metalloendopeptidase inhibitor activity, which is unsurprising given the ability of E2 in energy balance (36, 40).

Classically, the positive and negative feedback effects of E2 in LH have been both reported in teleosts. Negative feedback was documented in many species including salmonids, cyprinids, silurids, and perciformes (41). However, there were also lines of evidence in sexually immature teleosts for a positive feedback of estrogen on LH content and release, such as zebrafish (5, 6), croaker (8), Japanese eel (9), and goldfish (10). In this study, we also found that E2 could induce LH secretion and mRNA expression in prepubertal grass carp pituitary *in vivo* and *in vitro*. These results suggested that the estrogenic effect on LH expression during puberty mainly showed a pattern of positive feedback regulation (42–44), which is due to an inhibition of negative feedback regulation in this period (45).

Previous studies have reported that estrogen responsiveness of the pituitary gland requires the presence of nERs, including the classical ERα and ERβ (34). Given that (1) ERα, ERβ1, and ERβ2 were all highly detected in grass carp pituitary, (2) E2-induced LH secretion and mRNA expression could be blocked by nER antagonist (ICI182780), and (3) the ERβ agonist (DPN) could mimic the stimulated effect of E2 on LH secretion and mRNA expression, it would be logical to assume that E2-induced LH responses are mediated by ERβ in grass carp pituitary. In addition to the nERs, recent studies indicated that GPER was involved in suppressing GnRH-stimulated LH release in primary bovine pituitary cell (19). Furthermore, in mammals, GPER has been identified in the membrane of various target tissues, including pituitary (17, 18), which suggested that GPER may play a role in mediating the non-genomic effects of estradiol in the pituitary. However, to date, there are no studies showing GPER-mediated non-genomic signaling events in the teleost pituitary. In this study, GPER agonist (G-1) could induce LH secretion and LHβ mRNA expression in grass carp pituitary cells. Furthermore, GPER antagonist (G-15) could block E2-induced LHβ mRNA expression and LH secretion. These results, taken together, suggested that E2 could also activate GPER to induce LH secretion and mRNA expression in prepubertal grass carp pituitary.

Regarding the signal transduction for LH responses, E2-induced LH release and LHβ mRNA expression could be abolished by blocking the AC/cAMP/PKA pathway with AC or PKA inhibitors, inactivating the PLC/IP3 pathway with PLC or IP3 receptor blockers, or inhibiting the Ca^2+^/CaM/CaMK-II pathway with VSCC blocker, CaM antagonist, and CaMK-II inactivator. Collectively, our results imply that E2 can upregulate LH synthesis and secretion in grass carp pituitary cells *via* GPER coupled with AC/cAMP/PKA, PLC/IP3, and Ca^2+^ cascades. These findings are in agreement with the previous reports that E2 could rapidly activate different pathways including the stimulation of AC, mobilization of intracellular calcium (Ca^2+^) stores, and activation of mitogen-activated protein kinase and phosphoinositide 3-kinase signaling pathways (46, 47).

Concerning steroid feedback on FSH, the situation of E2 in teleosts is unclear and contradictory. Previous studies reported a negative effect of estradiol on FSH synthesis in salmonids (48, 49), whereas estradiol treatment could induce FSHβ mRNA expression in goldfish *in vivo* (50) and in eel *in vitro* (41). In this study, we demonstrated that the E2 could induce FSH secretion and synthesis in prepubertal grass carp pituitary *in vivo* and *in vitro*. Furthermore, by using several agonists and antagonists, we further confirmed that E2 could act through ERβ or GPER to induce FSH secretion and synthesis in grass carp pituitary cells. Similar to the regulation of LH, signal transduction studies indicated that E2 could induce FSHβ mRNA expression *via* GPER coupled with AC/cAMP/PKA, PLC/IP3, and Ca^2+^ cascades in grass carp pituitary cells.

In this study, E2 could also induce GREB1 mRNA expression in grass carp pituitary cells in a time-course and dose-dependent manner. In mammals, GREB1 is shown to be a key estrogen-specific ER-associated protein, where it is functionally linked with the transcriptional output of the ER complex (51). In teleosts, recent study revealed that GREB1 was expressed mainly in the pituitary and plays an important role in convergent extension movement and pituitary development in zebrafish (5, 6). In addition, GREB1 knockout could cause a reduction in LH and FSH secretion and mRNA expression in zebrafish (5, 6). In this study, we have also reported that E2 could induce LH and FSH secretion and synthesis in grass carp pituitary cells. These results, as a whole, suggested that E2-induced GREB1 mRNA expression played an important role in pituitary development and gonadotropin hormone expression in prepubertal grass carp pituitary.

**Figure 8:**
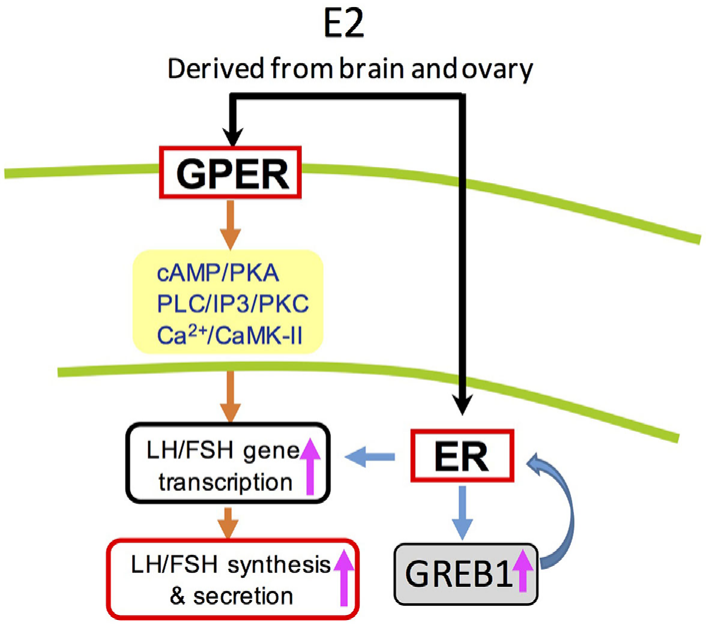
Working model of luteinizing hormone (LH) and follicle-stimulating hormone (FSH) regulation by E2 in grass carp pituitary cells. In grass carp pituitary cells, E2 could activate both protein-coupled estrogen receptor (GPER) and ER to induce LH and FSH secretion and synthesis. These effects appear to be mediated through the adenylyl cyclase/cAMP/protein kinase A, phospholipase C (PLC)/inositol 1,4,5-triphosphate/protein kinase C (PKC), and Ca^2+^/calmodulin/CaM-dependent protein kinase II (CaMK-II) cascades.

In summary, three nERs (ERα, ERβ1, and ERβ2) and two GPERs (GPER1a and GPER1b) were cloned and found to be highly expressed in grass carp hypothalamic-pituitary axis. Based on transcriptomic analysis, E2 could significantly regulate 64 genes expression in prepubertal grass carp pituitary cells, which were involved in reproductive hormone, gonad development, central nervous system development, cell proliferation, steroid receptor binding, calcium regulation, and energy balance. *In vivo* studies demonstrated that E2 could not only induce LH and FSH secretion in grass carp serum, but also upregulate FSHβ and LHβ mRNA expression in grass carp pituitary. For the *in vitro* studies, E2 could induce LH and FSH secretion and synthesis in grass carp pituitary cells. Receptor specificity showed that E2 could induce LH and FSH secretion and mRNA expression *via* activation of ERβ. Furthermore, the regulation of LH and FSH by E2 could also been *via* GPER1 coupled with AC/cAMP/PKA, PLC/IP3/PKC, and Ca^2+^/CaM/CaMK-II pathways. In addition to LH and FSH, E2 could also induce GREB1 (a novel regulator for pituitary development) mRNA expression in grass carp pituitary cells in a time-course and dose-dependent manner (Figure 8). These results, taken together, suggested that E2 could play an important role in inducing gonadotropin hormone (LH and FSH) release and pituitary development in prepubertal grass carp.

## Author Contributions

XL and GH conceived the project. XQ, YX, and CY performed the *in vitro* experiments. HL and GZ contributed to *in vivo* experiment. JJ and GH conducted the bioinformatics analysis. XL and GH contributed to the manuscript preparation.

## Conflict of Interest Statement

The authors declare that the research was conducted in the absence of any commercial or financial relationships that could be construed as a potential conflict of interest.

## Footnotes

^1^http://swissmodel.expasy.org/ (Accessed: June 1, 2019).

^2^http://www.ncbi.nlm.nih.gov/Traces/sra (Accessed: June 1, 2019).

^3^http://bioinfo.ihb.ac.cn/gcgd/php/index.php (Accessed: June 1, 2019).

^4^http://rsb.info.nih.gov/ij/index.html (Accessed: June 1, 2019).

## Abbreviations

AC, adenylyl cyclase; CaM, calmodulin; CaMK-II, CaM-dependent protein kinase II; IP3, inositol 1,4,5-triphosphate; PKC, protein kinase C; PKA, protein kinase A; PLC, phospholipase C; E2, 17β-estradiol; LH, luteinizing hormone; FSH, follicle-stimulating hormone; GREB1, growth regulation by estrogen in breast cancer 1.
