# Supplementary material for: Pituitary Action of E2 in Prepubertal Grass Carp: Receptor Specificity and Signal Transduction for Luteinizing Hormone and Follicle-Stimulating Hormone Regulation
Source: Front Endocrinol (Lausanne). 2018 Jun 8;9:308. doi: 10.3389/fendo.2018.00308 (PMC6002485; doi:10.3389/fendo.2018.00308)
Supplement: Supplementary file 1 [file presentation_1.PDF]

Supplementary Fig. 1

GPER1a

```
1     ATGGAAGAGCAGACTACCAC TGTGATTCCGATTTACTTGAATGGCACTGAGCAGTTCAAT
       M  E  E  Q  T  T  T  V  I  P  I  Y  L  N  G  T  E  Q  F  N
61    GCTTCATTTGAATTCAACCTAACCGATGTAAACGAAAGCATAGACACCTATGAGTTTTAC
       A  S  F  E  F  N  L  T  D  V  N  E  S  I  D  T  Y  E  F  Y
121   GTCATCGGCCTGTTTCTCTCCTGCCTGTACACCATATTCCTATTCCCCATTGGCTTCATT
       V  I  G  L  F  L  S  C  L  Y  T  I  F  L  F  P  I  G  F  I
181   GGCAACATCCTCATTTTAGTGGTCAACCTCAATCACAGGGACAAGATGACCATCCCTGAT
       G  N  I  L  I  L  V  V  N  L  N  H  R  D  K  M  T  I  P  D
241   CTGTACTTCGTCAACCTGGCCGTGGCGGACCTTATTCTAGTGGCGGATTCGCTCATTGAG
       L  Y  F  V  N  L  A  V  A  D  L  I  L  V  A  D  S  L  I  E
301   GTCTTCAATCTCAACGAGAAGTACTACGACTATGCCGTCCTCTGTACCTTCATGTCACTT
       V  F  N  L  N  E  K  Y  Y  D  Y  A  V  L  C  T  F  M  S  L
361   TTCCTCCAGGTGAACATGTACAGCAGCATCTTCTTCCTGACATGGATGAGTTTCGACCGC
       F  L  Q  V  N  M  Y  S  S  I  F  F  L  T  W  M  S  F  D  R
421   TATGTCGCTCTTGCCAGCTCCATTAGCAGCAGTCCGCTTCGAACTATGCAGCACGCCAAA
       Y  V  A  L  A  S  S  I  S  S  S  P  L  R  T  M  Q  H  A  K
481   CTCAGCTGCAGCCTCATCTGGATGGCCTCCATCCTGGCGACTCTGCTTCCCTTCACCATC
       L  S  C  S  L  I  W  M  A  S  I  L  A  T  L  L  P  F  T  I
541   GTGCAGACGCAACACACCGGCGAGGTGCACTTCTGCTTCGCCAATGTCTTGGAGATCCAG
       V  Q  T  Q  H  T  G  E  V  H  F  C  F  A  N  V  L  E  I  Q
601   TGGCTCGAGGTGACGATTGGATTTCTGGTGCCCTTCTCAATCATTGGCCTGTGCTATTCC
       W  L  E  V  T  I  G  F  L  V  P  F  S  I  I  G  L  C  Y  S
661   CTAATTGTCCGCATCCTCATGCGTGCCCAGAAGCACAAGGGACTGTGGCCGCGCCGGCAG
       L  I  V  R  I  L  M  R  A  Q  K  H  K  G  L  W  P  R  R  Q
721   AAGGCCCTGCGCATGATCGTGGTGGTTGTGCTGGTGTTCTTTATTTGCTGGCTGCCCGAG
       K  A  L  R  M  I  V  V  V  V  L  V  F  F  I  C  W  L  P  E
781   AACGTCTTCATTAGCATCCAGCTGCTCCAGGGCACGGCCGACCCATCGAAACGCAGCGAC
       N  V  F  I  S  I  Q  L  L  Q  G  T  A  D  P  S  K  R  S  D
841   ACCACGCTGTGGCACGACTACCCGTTGACCGGGCACATCGTCAACCTGGCTGCGTTCTCC
       T  T  L  W  H  D  Y  P  L  T  G  H  I  V  N  L  A  A  F  S
901   AACAGCTGCCTGAACCCGATCATTTACAGCTTCCTCGGGGAGACTTTCAGGGACAAGCTA
       N  S  C  L  N  P  I  I  Y  S  F  L  G  E  T  F  R  D  K  L
961   AGACTCTTCATCAAGAGGAAGGCCAGCTGGTCGGTGGTCTACCGCTTCTGTAATCACACT
       R  L  F  I  K  R  K  A  S  W  S  V  V  Y  R  F  C  N  H  T
1021  CTGGACTTGCACATCCCTGTCAGGGAACTTGATCAAGAAGTGACACGGGTTGACGTAACG
       L  D  L  H  I  P  V  R  E  L  D  Q  E  V  T  R  V  D  V  T
1081  AACACTGCTTAA
       N  T  A  *
```

Supplementary Fig. 1

GPER1b

```
1       ATGGAAGTGATACAGGGGAAG ATGA TGGA AGAT CCCA TGAC TTTT GACC CCAC CATC CAA
         M  E  V  I  Q  G  K  M  M  E  D  P  M  T  F  D  P  T  I  Q
61      CTGAATCAGTCCAGCCTGCCC TGGG TCAA TGAA ACGA ACTC CACC TCCC CGGA CACC TAC
         L  N  Q  S  S  L  P  W  V  N  E  T  N  S  T  S  P  D  T  Y
121     GCCATAAATCTTCTCCTCTCC TGCA TCTA CACC ATCC TGCT CTTC CCGC TTGG CCTT GTG
         A  I  N  L  L  L  S  C  I  Y  T  I  L  L  F  P  L  G  L  V
181     GGTAACATCCTGATACTGCTG GTGA ATTT CGAC CCTC GTCA GCGG ATGA GCAC CCCT GAT
         G  N  I  L  I  L  L  V  N  F  D  P  R  Q  R  M  S  T  P  D
241     CTATACTTCACTAATTTGGCT CTTG CCGA CCTG GTGC TGGT GCTG GACT CGCT AATC GAA
         L  Y  F  T  N  L  A  L  A  D  L  V  L  V  L  D  S  L  I  E
301     GTGTTCAATCTGAGTGCGCAC TACT ACGA TGAC GCAG TGCT GTGC TCCT GCAT GGCG ATC
         V  F  N  L  S  A  H  Y  Y  D  D  A  V  L  C  S  C  M  A  I
361     TTCCTGCAGGTCAACATGTAC AGCA GCGT GTTT TCGC TCAC CTGG ATGA GTCT GGAC CGC
         F  L  Q  V  N  M  Y  S  S  V  F  S  L  T  W  M  S  L  D  R
421     TGCCTTGCGCTGACTGGCCTC AGCA CACG TGCG CTAC CTGA AAAC GTTT CCGT CCAA CAC
         C  L  A  L  T  G  L  S  T  R  A  L  P  E  N  V  S  V  Q  H
481     CGTTCGCACATCGCCCGTAGG GCTT GCGC AACC ATTT GGGT GGCG GCGA CCCT GTGC ACG
         R  S  H  I  A  R  R  A  C  A  T  I  W  V  A  A  T  L  C  T
541     CTGATTCCATTCGCTACTGCG CACA TGTA TCAC GGCT GGGG GCGT GGCT TCTG TTTT GCG
         L  I  P  F  A  T  A  H  M  Y  H  G  W  G  R  G  F  C  F  A
601     GGTGTGGCCGAGGTGCAATGG CTGG AGGT GACG CTAG GATT CGCC CTTC CTTT TTGC GTG
         G  V  A  E  V  Q  W  L  E  V  T  L  G  F  A  L  P  F  C  V
661     ATGGGCGTCTGCTACACCCTG ATCG CACG CGTG CTGC TGCG CTCC GAAA GGCC GCAG CGA
         M  G  V  C  Y  T  L  I  A  R  V  L  L  R  S  E  R  P  Q  R
721     ATGGGCGTCTGCTACACCCTG ATCG CACG CGTG CTGC TGCG CTCC GAAA GGCC GCAG CGA
         T  K  A  L  H  M  I  V  A  A  V  S  V  F  F  V  C  W  L  P
781     GAGAACGTCTTCATCAGTGTG CATC TCTT GAGA GGCG ACAC AGAG GCCT CACG GCGG CGT
         E  N  V  F  I  S  V  H  L  L  R  G  D  T  E  A  S  R  R  R
841     GGAAACCACACGTTGTGGCAG CGCT ACCC ACTG ACGG GACA CGTG GTGA CCTT GGCG GCC
         G  N  H  T  L  W  Q  R  Y  P  L  T  G  H  V  V  T  L  A  A
901     TGCGCCAACAGCTGCCTGAAC CCGC TCGT CTAC AGCC TGCT GGGA AACA CCTT CAGA CAG
         C  A  N  S  C  L  N  P  L  V  Y  S  L  L  G  N  T  F  R  Q
961     AAACTGCAAGTGTTTATCGCG CACC ACGT GCGC TGCC TGCA TACA TGCA TGCA GAAC GCA
         K  L  Q  V  F  I  A  H  H  V  R  C  L  H  T  C  M  Q  N  A
1021    AGCGCGACGCCACCCTGTCCG TGCG TCAC TTGC ACAA ACGT GCAC CATT CCTG CTCG CAT
         S  A  T  P  P  C  P  C  V  T  C  T  N  V  H  H  S  C  S  H
1081    GAGAATGAGGAGGAGGAAGAG CGTG ACCT CAGG AGCG GCGA GGAG GGTC GAGA GTGT GTG
         E  N  E  E  E  E  E  R  D  L  R  S  G  E  E  G  R  E  C  V
1141    TGTGACAGAGTGGGATAA
         C  D  R  V  G  *
```

Supplementary Fig. 1

ERα

```
1      ATGTACCCTAAGGAGGAGCACAGCGTAGGAGCCATCAGCTCCTCTGTCAACTACATTGATGGAGCCTATGAGTACCCAGACCCCACACAG
        M  Y  P  K  E  E  H  S  V  G  A  I  S  S  S  V  N  Y  I  D  G  A  Y  E  Y  P  D  P  T  Q  30
91     ACCTACGGCACCTCATCACCTGCCTCTGTCGGATACTACCTGGCTCCCACGGACCCCCACGCACCCCCCGTTGAAGAACATCTGCAGACG
        T  Y  G  T  S  S  P  A  S  V  G  Y  Y  L  A  P  T  D  P  H  A  P  P  V  E  E  H  L  Q  T  60
181    TTGGGCGCTGGATCCAGCAGCCCCCTCATGTTTACACCCTCCAGCCCTCAGCTGTCCCCGTGCCTGAGCCATCATGGAGGACACCACTCC
        L  G  A  G  S  S  S  P  L  M  F  T  P  S  S  P  Q  L  S  P  C  L  S  H  H  G  G  H  H  S  90
271    ACCCACCAGGTGTCCTACTACCTGGACACATCTTCTAGCACAGTCTACAGGTCAGGTGTGGTATCTTCTCAGCAGCCAAGTGTTGGTCTG
        T  H  Q  V  S  Y  Y  L  D  T  S  S  S  T  V  Y  R  S  G  V  V  S  S  Q  Q  P  S  V  G  L  120
361    TGTGAAGTGTTGTGCAGTGCGACTGACAGGCAGGAGTTGTACACCGGATCAAGAGCTGCAGGAGGATTTGATTCAGGGAAGGAGACGCGC
        C  E  V  L  C  S  A  T  D  R  Q  E  L  Y  T  G  S  R  A  A  G  G  F  D  S  G  K  E  T  R  150
451    TTCTGTGCGGTGTGCAGTGACTATGCCTCTGGCTATCATTATGGAGTCTGGTCCTGCGAGGGATGCAAAGCTTTCTTCAAGAGAAGCATT
        F  C  A  V  C  S  D  Y  A  S  G  Y  H  Y  G  V  W  S  C  E  G  C  K  A  F  F  K  R  S  I  180
541    CAAGGTCACAACGACTATGTTTGTCCAGCGACCAACCAGTGCACTATTGACAGAAACCGCAGGAAGAGCTGCCAAGCATGCAGACTACGC
        Q  G  H  N  D  Y  V  C  P  A  T  N  Q  C  T  I  D  R  N  R  R  K  S  C  Q  A  C  R  L  R  210
631    AAGTGTTATGAAGTTGGCATGATGAAAGGAGGTATTCGTAAGGACCGCGGTGGCCGCGCTATCAGGCGTGAGAGGAGGAGGAGCAGTAAT
        K  C  Y  E  V  G  M  M  K  G  G  I  R  K  D  R  G  G  R  A  I  R  R  E  R  R  R  S  S  N  240
721    GAGGCTCGTGACAAGAGCTACAATGAGCAGTCAAGCCGTGCTGCACTGAGGACAGCTACCCCTCAGGACAAGAGGAAGAAGAGCAGCAGC
        E  A  R  D  K  S  Y  N  E  Q  S  S  R  A  A  L  R  T  A  T  P  Q  D  K  R  K  K  S  S  S  270
811    GGGGTGGCCAGCGCTATATTAATGCCACCTGACCAGGTGCTGGTGTTGCTTCTAGGGGCAGAGCCGCCGGCTGTCTGTTCACGTCAGAAG
        G  V  A  S  A  I  L  M  P  P  D  Q  V  L  V  L  L  L  G  A  E  P  P  A  V  C  S  R  Q  K  300
901    CACAGCCGCCCGTACACCGAGATCACCATGATGTCCCTGCTCACAAACATGGCTGACAAAGAACTCGTCCACATGATCGCCTGGGCTAAG
        H  S  R  P  Y  T  E  I  T  M  M  S  L  L  T  N  M  A  D  K  E  L  V  H  M  I  A  W  A  K  330
991    AAAGTACCAGGGTTCCAGGACCTCTCTCTGCATGACCAGGTTCAGTTGTTGGAGAGCTCTTGGCTGGAGGTGTTGATGATCGGCCTCATA
        K  V  P  G  F  Q  D  L  S  L  H  D  Q  V  Q  L  L  E  S  S  W  L  E  V  L  M  I  G  L  I  360
1081   TGGAGGTCCATTCATTCACCTGGAAAACTCATCTTTGCTCAGGATCTCATCCTTGATAGGAGTGAAGGAGAATGTGTTGAGGGAATGGCT
        W  R  S  I  H  S  P  G  K  L  I  F  A  Q  D  L  I  L  D  R  S  E  G  E  C  V  E  G  M  A  390
1171   GAGATTTTCGACATGCTTTTGGCGACTGTGGCTCGATTCCGCAGTCTCAAACTCAAGCTGGAGGAATTTGTTTGTCTTAAAGCCATCATA
        E  I  F  D  M  L  L  A  T  V  A  R  F  R  S  L  K  L  K  L  E  E  F  V  C  L  K  A  I  I  420
1261   CTTCTCAATTCTGGTGCATTTTCATTCTGCTCCAGTCCAGTGGAGCCCCTGATGGACAGCTTCATGGTGCAGTGCATGCTGGACAACATC
        L  L  N  S  G  A  F  S  F  C  S  S  P  V  E  P  L  M  D  S  F  M  V  Q  C  M  L  D  N  I  450
1351   ACTGATGCCCTCATTTACTGCATCAGTAAATCAGGTGCCTCGCTGCAGCTGCAGTCCCGCCGTCAGGCACAGCTCCTGCTGCTGCTCTCC
        T  D  A  L  I  Y  C  I  S  K  S  G  A  S  L  Q  L  Q  S  R  R  Q  A  Q  L  L  L  L  L  S  480
1441   CACATCAGACACATGAGCAACAAAGGAATGGAGCACTTATACCATATGAAATGTATGAATCGAGTCCCACTATATGATCATTTGCTGGAG
        H  I  R  H  M  S  N  K  G  M  E  H  L  Y  H  M  K  C  M  N  R  V  P  L  Y  D  H  L  L  E  510
1531   ATGCTGGATGCCCAACGATTCCAATCCCCAGGAAAGCTGCAGCAACTATGGGAACAGAGTGAGAAAGACCCCCCGTCTACACCAACAACC
        M  L  D  A  Q  R  F  Q  S  P  G  K  L  Q  Q  L  W  E  Q  S  E  K  D  P  P  S  T  P  T  T  540
1621   AGCAGCAGCAGCAGCCCCTCCAGAGGACCTGGAGCCATGCAGCCCAACACTGCCTGTCTCAGCCCAGACCCCTGA
        S  S  S  S  S  P  S  R  G  P  G  A  M  Q  P  N  T  A  C  L  S  P  D  P  *              564
```

Supplementary Fig. 1

ERβ1

```
1       ATGAGCTCCTCCCCTGATCCAGCCCCTACGTCAGTCTCCCCTGTCCTGGACTCCGGCAAGGCTGATCGAGGGGAC
         M  S  S  S  P  D  P  A  P  T  S  V  S  P  V  L  D  S  G  K  A  D  R  G  D   25
76      TCGCCTAAACTCTTACCCCATCTGTACACTTCCCCACTTGGCATGGATAACCAGACCATCTGCATTCCATCTCCG
         S  P  K  L  L  P  H  L  Y  T  S  P  L  G  M  D  N  Q  T  I  C  I  P  S  P   50
151     TATTTGGAAGCCTGTCAGGATTATTCACCACCGCACGGAGGAGAGTTTAACCATGGAGCTTTAACTCTGTACAGC
         Y  L  E  A  C  Q  D  Y  S  P  P  H  G  G  E  F  N  H  G  A  L  T  L  Y  S   75
226     CCGGTTTCCTCCTCAGTGCTAGGGTACCCTCATCCCCCCGTGTCTGAAAGCCTAGTCCCGCTCAGTCCCACAGTC
         P  V  S  S  S  V  L  G  Y  P  H  P  P  V  S  E  S  L  V  P  L  S  P  T  V   100
301     TTCTGGCCTTCCCACACCACACACACTGCGCTGTCTCTGCACTGCCCACCTCCCCTGGCCTACAGCGAAACACAC
         F  W  P  S  H  T  T  H  T  A  L  S  L  H  C  P  P  P  L  A  Y  S  E  T  H   125
376     TCACACACTGCCTGGGACGATGCCAAGAAACACACGCTTAACCAGAACAGTTCTGTCCTTACTCATGCAAAGCTG
         S  H  T  A  W  D  D  A  K  K  H  T  L  N  Q  N  S  S  V  L  T  H  A  K  L   150
451     TTAGGGCAGCAAGTGGAAGGTGATGATGGCTTGAATCCTTCACCAGGCATTGTGGGTAAAGGAGACACACACTTC
         L  G  Q  Q  V  E  G  D  D  G  L  N  P  S  P  G  I  V  G  K  G  D  T  H  F   175
526     TGTGCGGTGTGTCATGACTACGCCTCTGGGTATCACTATGGTGTCTGGTCATGTGAGGGGTGCAAGGCTTTCTTC
         C  A  V  C  H  D  Y  A  S  G  Y  H  Y  G  V  W  S  C  E  G  C  K  A  F  F   200
601     AAACGGAGCATTCAAGGACACAATGACTATATTTGTCCAGCCACCAACCAGTGCACCATTGACAAGAGCCGACGC
         K  R  S  I  Q  G  H  N  D  Y  I  C  P  A  T  N  Q  C  T  I  D  K  S  R  R   225
676     AAGAGCTGCCAGGCCTGTCGACTCCGCAAGTGCTATGAAATGGGCATGATGAAGTGTGGTGTGAGGCGGGAACGC
         K  S  C  Q  A  C  R  L  R  K  C  Y  E  M  G  M  M  K  C  G  V  R  R  E  R   250
751     TGCAGTTACCGAGGTGCTCGTCATCGCCGCAACCCCCAAATCAGAGACAGCTCGGGTGGGGCGTTAGGGGTCAGA
         C  S  Y  R  G  A  R  H  R  R  N  P  Q  I  R  D  S  S  G  G  A  L  G  V  R   275
826     GGTTGTTCCCAGCATCATTTAGAAGTTCCTCTCAATCCCACTCATCACCTCTTCCCTTCAGGGGGCAGAGCTGAG
         G  C  S  Q  H  H  L  E  V  P  L  N  P  T  H  H  L  F  P  S  G  G  R  A  E   300
901     GGGCGTGGCCTGAGCTTCTCCCCTGAGCAGTTGGTGAACTGTATTCTGGAGGCGGAGCCTCCTCAGATTTGCCTG
         G  R  G  L  S  F  S  P  E  Q  L  V  N  C  I  L  E  A  E  P  P  Q  I  C  L   325
976     AGAGAGCCAATGAAGAAGCCGTACACGGAGGCCAGCATGATGATGTCACTCACCAGCCTCGCTGACAAGGAACTG
         R  E  P  M  K  K  P  Y  T  E  A  S  M  M  M  S  L  T  S  L  A  D  K  E  L   350
1051    GTGCTCATGATCAGCTGGGCCAAGAAGATACCAGGTTTTGTGGAGCTGACACTTTCAAATCAGGTACATCTATTG
         V  L  M  I  S  W  A  K  K  I  P  G  F  V  E  L  T  L  S  N  Q  V  H  L  L   375
1126    GAATGCTGCTGGCTGGATATTCTGATGTTGGGATTGATGTGGAGATCTGTGGATCATCCCGGGAAACTCATCTTC
         E  C  C  W  L  D  I  L  M  L  G  L  M  W  R  S  V  D  H  P  G  K  L  I  F   400
1201    TCACCTGACCTCAAACTGAACAGGGATGAATGGAATTGTGTTGAAGGCATCATGGAGATCTTTGACATGCTGGTG
         S  P  D  L  K  L  N  R  D  E  W  N  C  V  E  G  I  M  E  I  F  D  M  L  V   425
1276    GCCACCACCTCCAGATTCAGAGAACTGAAGCTACAGAGGGAGGAATACGTCTGTCTCAAAGCCATGATCCTTCTC
         A  T  T  S  R  F  R  E  L  K  L  Q  R  E  E  Y  V  C  L  K  A  M  I  L  L   450
1351    AACTCTAATAACTGTTCAAGCTTGTCACAGACTCCTGAAGATGTGGAGAGTCGTGGGAAGGTTCTGAGGCTGCTG
         N  S  N  N  C  S  S  L  S  Q  T  P  E  D  V  E  S  R  G  K  V  L  R  L  L   475
1426    GACTCTGTAACTGACGCTCTGGTTTGGAGCATCTCCAGAACGGGCTTGTCCTCTCAGCAGCAGTCCATCCGGCTC
         D  S  V  T  D  A  L  V  W  S  I  S  R  T  G  L  S  S  Q  Q  Q  S  I  R  L   500
1501    GCCCATCTGCTGATGCTGCTTTCACACATTCGACACCTCAGCAACAAAGGCATTGAGCATCTGTCAAACATGAAA
         A  H  L  L  M  L  L  S  H  I  R  H  L  S  N  K  G  I  E  H  L  S  N  M  K   525
1576    AGAAAAAACGTGGTGCTGCTGTATGATCTTCTGCTGGAGATGCTGGACGCCAACACGTCCCAGAGCAACCGGATG
         R  K  N  V  V  L  L  Y  D  L  L  L  E  M  L  D  A  N  T  S  Q  S  N  R  M   550
1651    CTGGCGGCTCACACAGAAGCCTCTCTCCGGTCAGACACACAACAGACCCTCCACACATCCAGACCGCAGCCTGCA
         L  A  A  H  T  E  A  S  L  R  S  D  T  Q  Q  T  L  H  T  S  R  P  Q  P  A   575
1726    CCGAGAGAGAGAGACCAGGAGACCCGGCACAGTCCACAAGCTGAGGAGACATTGCACTCTGGTCATCATCGAGAG
         P  R  E  R  D  Q  E  T  R  H  S  P  Q  A  E  E  T  L  H  S  G  H  H  R  E   600
1801    GACATGGAGACAGACTGA
         D  M  E  T  D  *                                                            605
```

Supplementary Fig. 1

ERβ2

```
1      ATGTCCGAGTATCCAGAGGGAGAAAGCCCTCTGCTTCAGCTACAGGAAGTGGACTCCAGCAGGGTGGGAGGTCAC
        M  S  E  Y  P  E  G  E  S  P  L  L  Q  L  Q  E  V  D  S  S  R  V  G  G  H   25
76     GTCCTCTCACCTATCTTTAACTCCTCCTCTCCATCCCTGCCAGCGGAGAGCCATCCCATCTGTATCCCATCACCC
        V  L  S  P  I  F  N  S  S  S  P  S  L  P  A  E  S  H  P  I  C  I  P  S  P   50
151    TACACAGACCTCAGCCATGACTTCACCACTCTGCCCTTCTACAGTCCCGCTCTGCTGGGTTACGGCACATCGCCT
        Y  T  D  L  S  H  D  F  T  T  L  P  F  Y  S  P  A  L  L  G  Y  G  T  S  P   75
226    CTGTCCGACTGCTCATCAGTGCGGCAGTCGCTAAGCCCTACCCTCTTTTGGCCACCTCATAGCCAAGTTTCATCA
        L  S  D  C  S  S  V  R  Q  S  L  S  P  T  L  F  W  P  P  H  S  Q  V  S  S   100
301    CTTGCATTACATCAACAACAGACTCGATTACAACAAAACCATCCAACTGGTGGGACTTGGGCGGAACTTACACCA
        L  A  L  H  Q  Q  Q  T  R  L  Q  Q  N  H  P  T  G  G  T  W  A  E  L  T  P   125
376    TATGATCACGGCGAAGAGGAATACAGAAAACCACTGGTGAAGAGGGTAGCAGATTCAGAAGAGACTTCTACCTCT
        Y  D  H  G  E  E  E  Y  R  K  P  L  V  K  R  V  A  D  S  E  E  T  S  T  S   150
451    TCGAGAGGCAAAGCTGACATGCACTACTGTGCCGTTTGTAGTGATTATGCCTCTGGGTACCATTATGGTGTGTGG
        S  R  G  K  A  D  M  H  Y  C  A  V  C  S  D  Y  A  S  G  Y  H  Y  G  V  W   175
526    TCGTGTGAAGGATGCAAAGCCTTCTTCAAGAGGAGTATACAAGGACACAATGACTACATCTGCCCTGCCACCAAC
        S  C  E  G  C  K  A  F  F  K  R  S  I  Q  G  H  N  D  Y  I  C  P  A  T  N   200
601    CAGTGCACCATCGACAAGAACCGCCGCAAAAGCTGCCAGGCCTGCCGACTCCGGAAGTGTTATGAAGTTGGAATG
        Q  C  T  I  D  K  N  R  R  K  S  C  Q  A  C  R  L  R  K  C  Y  E  V  G  M   225
676    ATGAAATGTGGGTTACGGCGAGATCGTGGCAGCTACCAACAAAGAGGAGCACAACAGAAGCGAATGGCACGATTC
        M  K  C  G  L  R  R  D  R  G  S  Y  Q  Q  R  G  A  Q  Q  K  R  M  A  R  F   250
751    TCTGGCAGGATGAGAACAAGTGGCCCGAAATCTCAAGAGATGAAAAGTGTCCCACGTCCCCTCAGTGGAAATGAG
        S  G  R  M  R  T  S  G  P  K  S  Q  E  M  K  S  V  P  R  P  L  S  G  N  E   275
826    GTGGTTACCATGGCGTTGAGCCCTGAGCAACTAATCGCTCGCATCATGGATGCAGAGCCACCTGAGATTTACCTC
        V  V  T  M  A  L  S  P  E  Q  L  I  A  R  I  M  D  A  E  P  P  E  I  Y  L   300
901    ATGAAAGATGTGAAGAAGCCATTTACTGAGGCCAACGTCATGATGTCACTGACCAACCTAGCTGACAAAGAGCTC
        M  K  D  V  K  K  P  F  T  E  A  N  V  M  M  S  L  T  N  L  A  D  K  E  L   325
976    GTTCACATGATCAGCTGGGCCAAGAAGATCCCAGGTTTTGTGGAGCTCAGTCTTTTTGACCAGGTCCATTTGTTA
        V  H  M  I  S  W  A  K  K  I  P  G  F  V  E  L  S  L  F  D  Q  V  H  L  L   350
1051   GAGTGCTGCTGGTTAGAGGTGCTGATGTTGGGACTAATGTGGCGTTCTGTTAATCACCCTGGAAAGCTCATTTTC
        E  C  C  W  L  E  V  L  M  L  G  L  M  W  R  S  V  N  H  P  G  K  L  I  F   375
1126   TCTCCAGACCTCAGTCTCAGCAGAGACGAAGGCAGCTGTGTGCAGGGGTTTGTGGAAATCTTTGATATGCTGCTG
        S  P  D  L  S  L  S  R  D  E  G  S  C  V  Q  G  F  V  E  I  F  D  M  L  L   400
1201   GCAGCCACGTCCAGATTCAGAGAACTCAAATTACAGAGAGAGGAGTATGTGTGTCTCAAAGCCATGATCCTCCTC
        A  A  T  S  R  F  R  E  L  K  L  Q  R  E  E  Y  V  C  L  K  A  M  I  L  L   425
1276   AACTCCAACATGTGTCTGAGTTCGTCTGAGGGAGGAGAGGAACTGCAGAGCCGGTCGAAGCTGCTGTGTCTGCTG
        N  S  N  M  C  L  S  S  S  E  G  G  E  E  L  Q  S  R  S  K  L  L  C  L  L   450
1351   GACTCGGTGACGGATGCTCTGGTATGGGCCATCTCGAAGACAGGCTTGAGCTTTCAGCAGCGCTCCACCAGGCTA
        D  S  V  T  D  A  L  V  W  A  I  S  K  T  G  L  S  F  Q  Q  R  S  T  R  L   475
1426   GCCCATCTGCTCATGCTGCTCTCCCACATAAGGCACCTCAGTAACAAAGGCATGGACCACCTCCACTGCATGAAG
        A  H  L  L  M  L  L  S  H  I  R  H  L  S  N  K  G  M  D  H  L  H  C  M  K   500
1501   ATGAAGAAGATGGTTCCTCTGTATGACCTGCTTCTGGAGATGCTGGACGCTCATATCATGCACAGCTCACGTCTG
        M  K  K  M  V  P  L  Y  D  L  L  L  E  M  L  D  A  H  I  M  H  S  S  R  L   525
1576   TCTCACTCCAGTCCACGAGAGAGCAAAGGCGTCCAGGAGGCCTTCATCTGCACTTCTCAGCATGGACCCTAA
        S  H  S  S  P  R  E  S  K  G  V  Q  E  A  F  I  C  T  S  Q  H  G  P  *      548
```

Supplemental Fig. S1. Nucleotide and deduced amino acid sequences of the grass carp ERα , ERβ1, ERβ2, GPER1a and GPER1b.

Supplementary Fig. 2

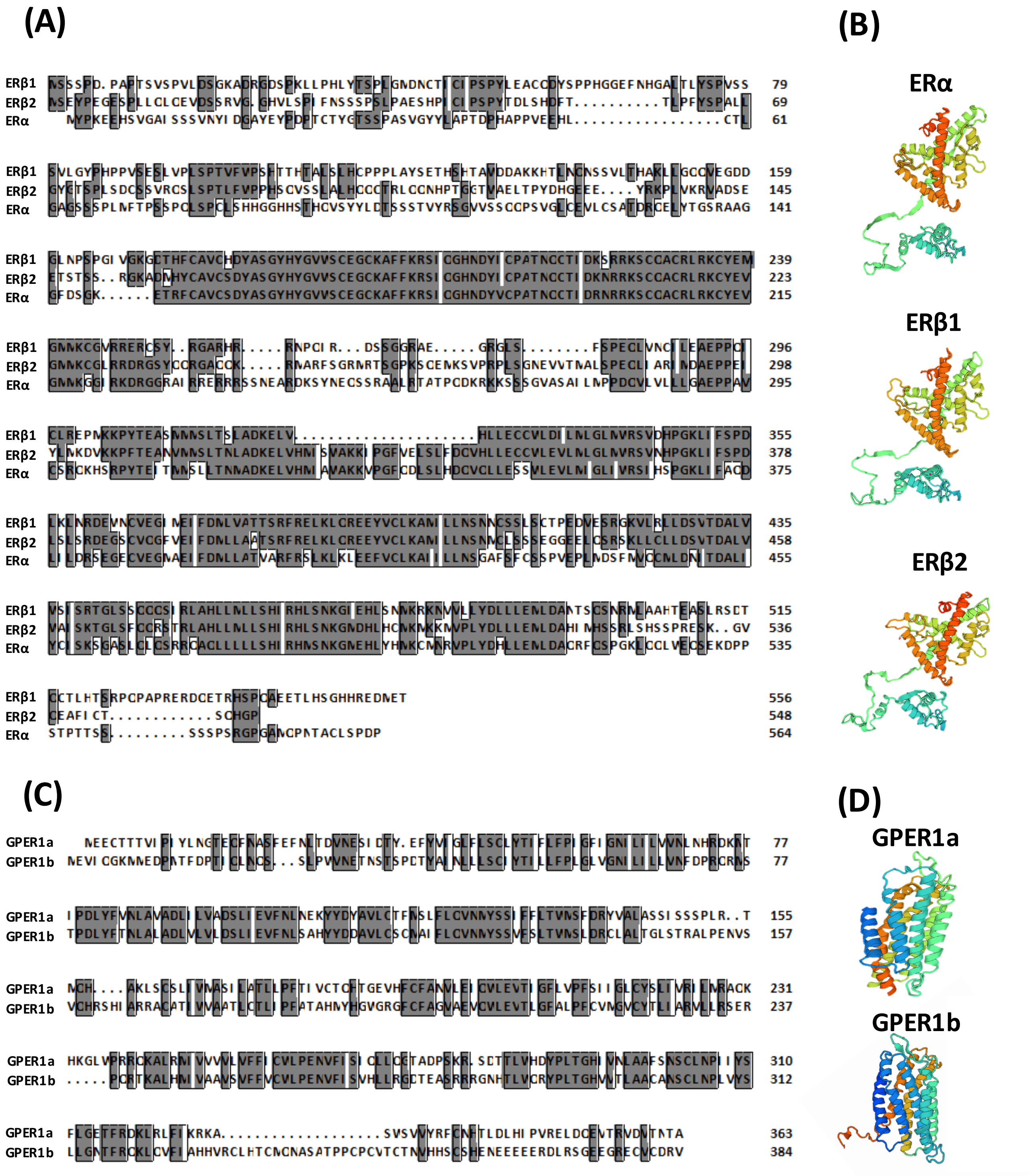

Supplemental Fig.S2. Sequence analysis of grass carp nERs and GPERs. (A) Amino acid alignment among grass carp ERα , ERβ1 and ERβ2 using Clustal-W algorithm with Mac Vector program. (B) 3-D protein model of grass carp ERα , ERβ1 and ERβ2 was deduced based on the crystal structure of human ER using SWISS-MODEL program. (C) Protein sequence alignment between grass carp GPER1a and GPER1b using Clustal-W algorithm with Mac Vector program. (D) 3-D protein model of grass carp GPER1a and GPER1b was deduced using SWISS-MODEL program.

Supplementary Fig.3

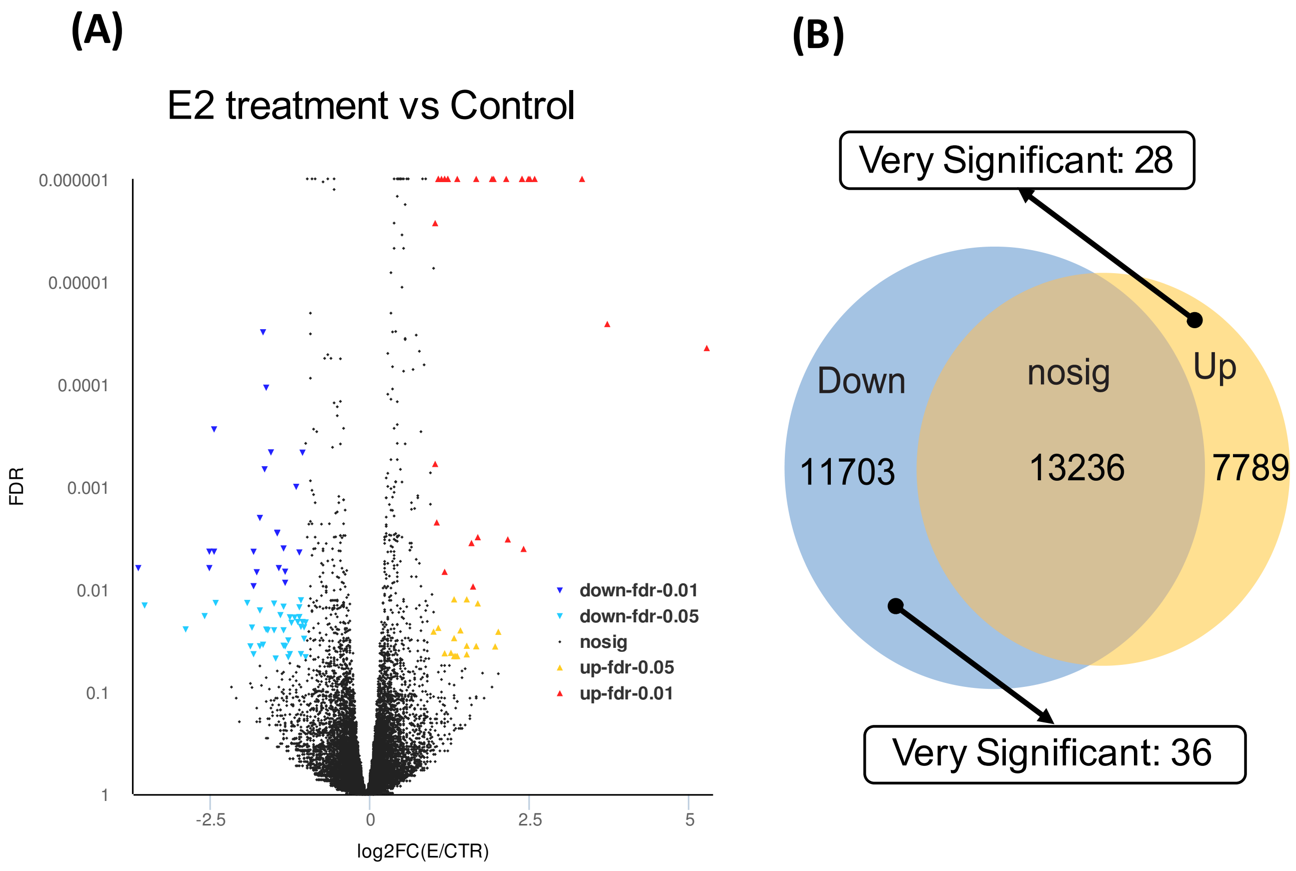

Supplementary Fig.3 Number of genes regulated by E2 in grass carp pituitary cells. (A) Volcano plot of DEGs between the E2 treatment and control group in grass carp pituitary cells. The red and blue dots expressed the up- and down-regulated genes, respectively. The black dots indicated the genes without significantly differential expression. (B) Venn diagram showing overlap of genes regulated by E2 in grass carp pituitary cells.
