# Supplementary material for: Pituitary Action of E2 in Prepubertal Grass Carp: Receptor Specificity and Signal Transduction for Luteinizing Hormone and Follicle-Stimulating Hormone Regulation
Source: Front Endocrinol (Lausanne). 2018 Jun 8;9:308. doi: 10.3389/fendo.2018.00308 (PMC6002485; doi:10.3389/fendo.2018.00308)
Supplement: Supplementary file 2 [file table_1.docx]

|  | Forward primer | Reverse primer | Annealing Tm(℃) | Product size (bp) |
| --- | --- | --- | --- | --- |
| ERα | AGAGAAGCATTCAAGGTCAC | TGTCACGAGCCTCATTACTG | 50 | 204 |
| ERβ1 | AAGGCATTGAGCATCTGTC | TCTGTCTCCATGTCCTCTC | 51 | 269 |
| ERβ2 | CGTCCAGATTCAGAGAACT | GGTCCATGCTGAGAAGTG | 51 | 436 |
| GPER1a | ACTACGACTATGCCGTCCTCTG | CACCAGAAATCCAATCGTCACC | 53 | 308 |
| GPER1b | GGAAGATGATGGAAGATCC | CTGCGTCATCGTAGTAGT | 50 | 321 |
| LHβ | GCTCAAAGCTCTTTTCTTCCACCA | GCTGCAGGCTTTCGATGGTACAG | 56 | 319 |
| FSHβ | TTCGTTGTTATGGTGATGCT | CGTGAAAACCGAGTCAGTCC | 52 | 282 |
| GREB1 | TGCTCTGCTCTCACTGTA | ACCTCTTCCTCCTCATTGT | 50 | 356 |
| β-actin | CTGGTATCGTGATGGACTCT | AGCTCATAGCTCTTCTCCAG | 56 | 285 |

**Supplemental Table 1.** Primer sequences and PCR conditions for real-time PCR for selected gene targets in grass carp.
