# Supplementary material for: Pituitary Action of E2 in Prepubertal Grass Carp: Receptor Specificity and Signal Transduction for Luteinizing Hormone and Follicle-Stimulating Hormone Regulation
Source: Front Endocrinol (Lausanne). 2018 Jun 8;9:308. doi: 10.3389/fendo.2018.00308 (PMC6002485; doi:10.3389/fendo.2018.00308)
Supplement: Supplementary file 3 [file table_2.docx]

**Supplemental Table 2** Antibodies used in Western Blot and fluorescence immunoassay

| **Protein Target** | **Antigen and source of sequence information** | **Name of antibody** | **Name of individual providing the antibody** | **Species for Raising antibody/Application** |
| --- | --- | --- | --- | --- |
| **grass carp LH** | Recombinant grass carp LHβ (GenBank EF565171) | grass carp LH antibody | Dr. Hu GF, Huazhong Agricultural University | Polyclonal in Rabbit |
| **grass carp FSH** | Recombinant grass carp FSHβ (GenBank EF552359) | grass carp FSH antibody | Dr. Hu GF, Huazhong Agricultural University | Polyclonal in Rabbit |
| **β-actin** | A synthetic peptide (KLH coupled) convering the conserved region of human, rat, and mouse actin | Anti-actin mAb | Calbiochem, catlog#CP01 | Monoclonal IgM in Mouse |
